# Clerodane diterpenoids with anti-inflammatory activity from the tuberous root of *Paratinospora sagittata* (Oliv.) Wei Wang

**DOI:** 10.3389/fphar.2025.1561954

**Published:** 2025-03-20

**Authors:** Hailin Li, Jing Liu, Chuhong Fang, Shanglei Ning

**Affiliations:** ^1^ Department of Hepatobiliary Surgery, General Surgery, Qilu Hospital of Shandong University, Jinan, Shandong, China; ^2^ Shandong Laboratory of Yantai Drug Discovery, Bohai Rim Advanced Research Institute for Drug Discovery, Yantai, Shandong, China

**Keywords:** clerodane, diterpenoid, *Paratinospora sagittata*, *Tinospora sagittata*, anti-inflammatory activity

## Abstract

Seven novel clerodane diterpenoids, designated tinotanoids I–O (1−7), were isolated from the tuberous roots of *Paratinospora sagittata* (Oliv.) Wei Wang [syn.: *Tinospora sagittata* var. yunnanensis (S.Y.Hu) H.S.Lo; Menispermaceae]. The structural elucidation of these new metabolites, including their absolute configurations, was achieved through advanced spectroscopic methods such as mass spectrometry (MS), nuclear magnetic resonance (NMR), electronic circular dichroism (ECD), and X-ray crystallography. Evaluation of their anti-inflammatory activity revealed that metabolites 3 and 4 exhibited potent inhibition of nitric oxide (NO) production in lipopolysaccharide (LPS)-activated RAW264.7 macrophages, with IC_50_ values of 12.5 ± 0.5 and 16.4 ± 0.7 *μ*M, respectively, which are better than those of the positive controls, quercetin and dexamethasone. Additionally, metabolites 3 and 4 effectively inhibited the pro-inflammatory cytokines TNF-α and IL-6 in a dose-dependent manner.

## 1 Introduction

**FIGURE 1:**
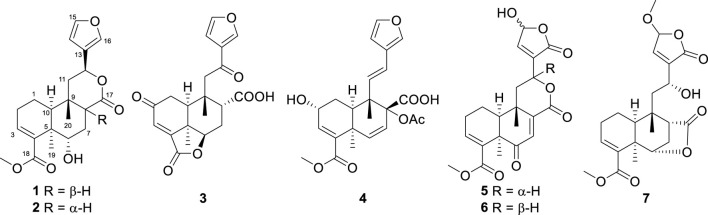
Structures of metabolites 1−7.

The *Tinospora* genus is abundant in clerodane diterpenoids (Zhang J.-S. et al., 2021; Zhang et al., 2022; Zhu et al., 2022; Zhu et al., 2023; Nishidono and Tanaka, 2024; Song J.-Q. et al., 2024), alkaloids (Huang et al., 2023; Liang et al., 2024), and lignans (Huang et al., 2012; Zhang J. S. et al., 2021), showcasing a wide array of biological activities, including anti-inflammatory (Zhu et al., 2023; Song Y.-Z. et al., 2024), antitumor (Mantaj et al., 2015; Zhang et al., 2022), antibacterial (Royani et al., 2023; Song J.-Q. et al., 2024), immunomodulation (Ao et al., 2024), and anti-diabetic (Lam et al., 2023; Mishra et al., 2023; Kabbashi et al., 2024) activities. *Tinospora sagittata*, one of the six Chinese native species of *Tinospora*, is widespread across southern China. Jin Guo Lan, a revered traditional Chinese medicine documented in each year’s edition of the Chinese Pharmacopoeia, is carefully crafted from the tuberous roots of *T. sagittata* and *T. capillipes*. These roots are highly valued for their efficacy in clearing heat and detoxifying the body, a property that has also been harnessed in the traditional ethnomedicine practiced by the “Dai” people (Huang et al., 2010; Zhang et al., 2016). In this study, we examined the metabolites of *T. sagittata*, sourced from Xishuangbanna, Yunnan Province. Our findings include the successful isolation and detailed characterization of seven clerodane diterpenoids from the plant (Figure 1), together with an assessment of their potential anti-inflammatory activity.

## 2 Materials and methods

### 2.1 General experimental procedures

X-ray diffraction data were acquired using an Agilent Xcalibur Nova X-ray diffractometer. Melting points were determined using an X-4 apparatus and reported without correction. Optical rotations were measured using a Perkin–Elmer 341 Polarimeter. NMR spectra were acquired using a Bruker AM-400 Spectrometer at 25°C. Electrospray ionization mass spectrometry (ESIMS) and high-resolution electrospray ionization mass spectrometry (HRESIMS) were performed using a Finnigan LCQ Deca system. Absorbance measurements were taken at 490 and 540 nm using a microplate reader (Molecular Devices, United States) and analyzed using SoftMax Pro 5 software (Molecular Devices, United States). For high-performance liquid chromatography (HPLC), a Shimadzu LC-20AT System coupled with an SPD-M20A PDA detector was used, and a YMC-pack ODS-A column (250 × 10 mm, S-5 μm, 12 nm) was used for semi-preparative HPLC. Column chromatography (CC) was conducted using silica gel (300–400 mesh, Qingdao Haiyang Chemical Co. Ltd.), reversed-phase C18 (RP-C18) silica gel (12 nm, S-50 μm, YMC Co. Ltd.), and MCI gel (CHP20P, 75–150 μm, Mitsubishi Chemical Industries Ltd.). All solvents were of analytical grade, sourced from Guangzhou Chemical Reagents Company, Ltd.

### 2.2 Plant materials

The tuberous roots of *Paratinospora sagittata* (Oliv.) Wei Wang [syn.: *Tinospora sagittata* var. yunnanensis (S.Y.Hu) H.S.Lo, Menispermaceae] were collected in August 2022 from Xishuangbanna, Yunnan province, China. The plant materials were authenticated by Jing Liu. A voucher specimen has been deposited at the Shandong Laboratory of Yantai Drug Discovery (accession number: JGL).

### 2.3 Extraction and isolation

The air-dried powder of the tuberous roots of *T. sagittata* (10 kg) was subjected to extraction by soaking in 95% ethanol at ambient temperature, with the process repeated three times at weekly intervals. Following this, the ethanol extract was concentrated to yield 900 g of dried material. This residue was then reconstituted by stirring in 5.0 L of water and subsequently subjected to liquid–liquid extraction with an equal volume of ethyl acetate in a separating funnel. The ethyl acetate layer was further fractionated using D101 macroporous resin column chromatography with a gradient of ethanol–water mixtures (30%, 60%, and 90%) to yield three distinct fractions. The 60% EtOH elution (140 g) was fractionated using silica gel CC and eluted with petroleum ether–EtOAc (5:1 to 0:1) to produce seven subfractions (A–G). Fraction E (10.0 g) was separated using silica gel CC (CH_2_Cl_2_–MeOH, 40:1 to 1:1) to produce eight subfractions (E1–E8), and fraction E5 was fractionated using RP-18 CC (MeOH–H_2_O, 30%–60%) to yield another eight subfractions (E4A–E4H). Fraction E4D was purified via Sephadex LH-20 column chromatography with methanol as the eluent, resulting in the isolation of metabolite 7 (40 mg). Fraction E4F was further separated using silica gel column chromatography with a gradient of petroleum ether and acetone (1:1 to 1:2) to yield four subfractions (E4F1–E4F4). Subsequently, subfractions E4F1 and E4F2 were purified using HPLC with the same solvent system (33% MeCN–H_2_O), leading to the isolation of metabolite 3 (2.2 mg; t_R_ = 19.5 min) and 4 (8.5 mg; t_R_ = 14.5 min), respectively. Fraction E6 was fractionated using silica gel CC (petroleum ether–acetone, 1:1 to 1:2) to yield three subfractions (E6A–E6C). Fraction E6B was purified using HPLC (36% MeCN–H_2_O) to produce 1 (4.0 mg; t_R_ = 14.5 min) and 2 (2.0 mg; t_R_ = 16 min), while E6C was also processed using HPLC (32% MeCN–H_2_O) to yield 5 (1.5 mg; t_R_ = 18.5 min) and 6 (2 mg; t_R_ = 19.0 min).

### 2.4 Spectroscopic data

Tinotanoid I (1): Colorless crystal, CCDC 2411568; Flack parameter, 0.10 (5); other crystal data are provided in Supplementary Table S1 in SI; m.p. 136−138°C; [*α*]^25^_D_ −180 (*c* 0.05, MeOH); UV (MeOH) λ_max_ (log ε), 209 nm (3.46) ; ECD (*c* 5.4 × 10^−4^ M, MeOH) *λ*_max_ (Δ*ε*), 239 nm (−11.49); ^1^H and ^13^C NMR data are provided in Tables 1, 2; HRESIMS *m*/*z* 375.1805 [M + H]^+^ (calcd for C_21_H_27_O_6_^+^, 375.1802).

**TABLE 1:** ^1^H NMR data of metabolites 1−7 in CDCl_3_ (recorded at 600 MHz; *δ* in ppm; *J* in Hz).

No.	1	2	3	4	5	6	7
1	2.07, m	2.08, m	2.93, dd (18.6, 6.9)	2.29, m	2.17, dt (15.6, 8.7)	2.12, td (15.6, 8.9)	1.94, m (2H)
	1.77, m	1.78, m	2.69, d (18.6)	1.85, m	1.89, m	1.95, m	
2	2.37, m	2.41, m		4.43, m	2.44, m (2H)	2.44, m (2H)	2.39, m
	2.32, m	2.34, m					2.31, m
3	6.77, t (3.6)	6.61, t (3.9)	6.66, s	6.70, brs	6.78, t (4.0)	6.76, t (4.0)	7.02, brt (3.5)
6	4.62, brd (4.4)	4.83, brs	5.08, dd (10.6, 7.3)	6.49, d (10.3)			5.51, d (6.1)
7	2.51, dt (14.4, 4.4)	2.04, m	2.53, m	6.32, d (10.3)	6.65, s	6.98, s	2.17, ddd (12.0, 6.1, 5.5)
	1.89, dd (14.4, 5.6)	1.66, ddd (14.2, 12.3, 1.7)	1.54, m				1.98, m
8	2.21, dd (5.6,4.4)	3.32, dd (12.1, 2.7)	3.22, t (3.5)				2.71, t (5.5)
10	2.28, brd (6.4)	2.08, m	2.65, d (6.9)	2.54, brd (5.6)	2.30, dd (6.7, 1.7)	2.01, dd (6.6, 1.0)	1.51, m
11	2.26, dd (14.3, 1.8)	2.08, dd (13.9, 6.1)	3.19, d (15.1)	6.14, d (16.3)	2.49, dd (14.5, 3.1)	2.63, dd (13.9, 3.0)	2.23, dd (15.1, 7.0)
	1.64, dd (14.3, 12.6)	1.95, dd (13.9, 11.4)	2.57, d (15.1)		1.82, dd (14.5, 12.0)	1.56, m	1.76, d (15.1)
12	5.55, dd (12.6, 1.8)	5.39, dd (11.4, 6.1)		6.22, d (16.3)	4.93, ddd (12.0, 3.1, 1.0)	5.42, ddd (12.1, 3.0, 1.0)	4.89, d (7.0)
14	6.44, brs	6.42, d (1.0)	6.75, d (1.3)	6.56, brs	7.31, dd (1.4, 1.0)	7.28, brs	7.03, s
15	7.41, brs	7.42, t (1.6)	7.48, t (1.3)	7.35, brs	6.22, brs	6.21, brs	5.28, s
16	7.49, brs	7.46, brs	8.10, brs	7.40, brs			
19	1.38, s	1.37, s	1.56, s	1.54, s	1.44, s	1.41, s	1.34, s
20	1.14, s	1.05, s	0.97, s	0.99, s	1.26, s	1.37, s	1.22, s
18-OMe	3.74, s	3.71, s		3.74, s	3.70, s	3.71, s	3.72, s
15-OMe							3.56, s
OAc				2.07, s			

**TABLE 2:** ^13^C NMR data of metabolites 1−7 in CDCl_3_ (151 MHz; *δ* in ppm).

No.	1	2	3	4	5	6	7
1	16.9	16.8	35.5	29.6	17.8	17.3	16.4
2	23.9	23.4	197.3	64.3	23.0	22.8	24.1
3	140.6	140.1	128.6	140.1	139.0	138.5	142.6
4	136.8	136.4	153.0	136.9	134.2	134.6	134.1
5	39.4	40.0	40.6	37.1	47.2	46.5	39.2
6	69.3	68.9	82.5	136.1	200.2	200.5	83.3
7	28.8	27.1	29.0	124.4	131.0	133.2	29.5
8	45.7	40.7	47.0	82.5	148.2	145.3	47.6
9	35.7	37.7	39.1	44.6	38.1	37.4	39.5
10	36.8	45.7	46.4	44.1	48.5	48.8	45.1
11	42.8	45.8	46.5	132.2	42.5	40.0	44.8
12	69.7	70.3	195.2	120.0	71.1	71.3	64.8
13	125.1	124.5	129.0	124.4	135.0	136.5	140.0
14	108.6	108.6	108.5	107.8	145.9	145.5	142.2
15	143.6	143.6	148.6	143.3	97.6	97.6	103.0
16	139.7	139.5	145.1	139.6	168.5	168.5	170.8
17	174.6	175.3	175.4	166.9	166.5	163.3	179.1
18	168.0	167.7	168.1	172.5	167.0	167.1	166.7
19	27.0	28.1	28.1	30.8	26.1	26.5	27.1
20	27.6	21.8	21.1	16.9	26.2	19.3	21.8
15-OMe							57.2
18-OMe	51.7	51.7		51.8	52.1	52.1	51.7
OAc				169.7			
				21.0			

Tinotanoid J (2): White solid; [*α*]^25^_D_ −33.1 (*c* 0.10, MeOH); UV (MeOH) λ_max_ (log ε), 208 nm (3.52); ECD (*c* 6.7 × 10^−4^ M, MeOH) *λ*_max_ (Δ*ε*), 241 nm (−4.13) and 214 nm (+2.95); ^1^H and ^13^C NMR data are listed in Tables 1, 2, respectively; HRESIMS *m*/*z* 375.1804 [M + H]^+^ (calcd for C_21_H_27_O_6_^+^, 375.1802).

Tinotanoid K (3): Colorless oil; [*α*]^25^_D_ −48.7 (*c* 0.30, MeOH); UV (MeOH) λ_max_ (log ε), 209 nm (3.61) and 240 nm (3.25); ECD (*c* 2.7 × 10^−4^ M, MeOH) *λ*_max_ (Δ*ε*), 281 nm (+6.78) and 232 nm (−7.79); ^1^H and ^13^C NMR data are listed in Tables 1, 2, respectively; HRESIMS *m*/*z* 373.1286 [M + H]^+^ (calcd for C_20_H_21_O_7_^+^, 373.1282).

Tinotanoid L (4): Colorless oil; [*α*]^25^_D_ −89.1 (*c* 0.20, MeOH); UV (MeOH) λ_max_ (log ε), 211 nm (3.81); ECD (*c* 2.3 × 10^−4^ M, MeOH) *λ*_max_ (Δ*ε*), 253 nm (−1.52) and 218 nm (+14.22); ^1^H and ^13^C NMR data are listed in Tables 1, 2, respectively; HRESIMS *m*/*z* 429.1551 [M − H]^−^ (calcd for C_23_H_25_O_8_^−^, 429.1555).

Tinotanoid M (5): Colorless oil; [*α*]^25^_D_ +11.5 (*c* 0.20, MeOH); UV (MeOH) λ_max_ (log ε), 206 nm (3.44); ECD (*c* 5.0 × 10^−4^ M, MeOH) *λ*_max_ (Δ*ε*), 268 nm (−2.14), 236 nm (+10.61), and 215 nm (−4.70); ^1^H and ^13^C NMR data are listed in Tables 1, 2, respectively; HRESIMS *m*/*z* 401.1244 [M − H]^−^ (calcd for C_21_H_21_O_8_^−^, 401.1242).

Tinotanoid N (6): Colorless oil; [*α*]^25^_D_ +8.7 (*c* 0.20, MeOH); UV (MeOH) λ_max_ (log ε), 207 nm (3.59); ECD (*c* 5.0 × 10^−4^ M, MeOH) *λ*_max_ (Δ*ε*), 268 nm (−2.12), 238 nm (+10.48), and 216 nm (−4.25); ^1^H and ^13^C NMR data are listed in Tables 1, 2, respectively; HRESIMS *m*/*z* 401.1243 [M − H]^−^ (calcd for C_21_H_21_O_8_^−^, 401.1242).

Tinotanoid O (7): Colorless oil; [*α*]^25^_D_ −30.6 (*c* 0.20, MeOH); UV (MeOH) λ_max_ (log ε), 211 nm (3.65); ECD (*c* 4.8 × 10^−4^ M, MeOH) *λ*_max_ (Δ*ε*), 249 nm (−1.85) and 215 nm (+2.64); ^1^H and ^13^C NMR data are listed in Tables 1, 2, respectively; HRESIMS *m*/*z* 443.1676 [M + Na]^+^ (calcd for C_22_H_28_O_8_Na^+^, 443.1676).

### 2.5 ECD and NMR calculation procedure

The absolute configurations of metabolites 2−4 were assigned by theoretical ECD calculation using the time-dependent density functional theory (TDDFT) approach. The initial conformations of the target molecules were generated using the MM2 force field within the ChemDraw Pro 14.1 software suite. Subsequent conformational searches were conducted using a hybrid torsional/low-mode sampling approach, employing MMFFs within an energy window of 2.58 kcal/mol. This was achieved through the conformational search module integrated into Maestro 10.2 software. The re-optimization of these conformations, followed by TD-DFT calculations on the refined structures, was executed using Gaussian 09 at the B3LYP/6-311G (d,p) level of theory in a vacuum. To ensure that the re-optimized conformers represented energy minima, a frequency analysis was also performed. Finally, SpecDis 1.64 software was used to generate the Boltzmann-weighted average ECD spectra.

NMR calculations for metabolite 4 were performed using the gauge-including atomic orbitals (GIAO) approach at the B3LYP/6-31G(d) level of theory, with the polarizable continuum model (PCM) used to simulate chloroform as the solvent. The NMR data for the lowest energy conformers of each metabolite were averaged based on the principles of Boltzmann distribution. The correlation between experimental and calculated NMR data was assessed using the enhanced probability method DP4+, and the results are provided in the accompanying supplementary data file.

### 2.6 Analysis of NO production

RAW264.7 macrophages were plated into 96-well plates at a density of 8 × 10^4^ cells/well for 24 h and then pre-incubated with varying concentrations of test metabolites for 1 h prior to stimulation with lipopolysaccharide (LPS) at a concentration of 0.1 *μ*g/mL for 24 h. The concentration of nitric oxide (NO) in the culture supernatant was then quantified using a Griess reagent kit. Absorbance (A) at 540 nm was measured using a multifunction microplate reader. The inhibition of cell growth was calculated according to the following formula: Inhibition (%) = [1 − (A_LPS + sample_ − A_untreated_) / (A_LPS_ − A_untreated_)] × 100. The experiments were performed in triplicates, and the data were presented as the mean ± SD. Quercetin was used as a positive control.

### 2.7 Cytotoxicity assay

The cytotoxicity of the isolated metabolites toward RAW264.7 cells was evaluated using an MTT assay. RAW264.7 cells were planted in 96-well plates (8 × 10^3^/well) for 24 h. Subsequently, the cells were exposed to the test metabolites, which were initially dissolved in DMSO and then diluted in DMEM to a final volume of 100 *μ*L per well, achieving a concentration of 50 *μ*M for the metabolites and maintaining a 1% DMSO concentration. Wells without cells containing only 100 *μ*L of DMEM served as the blank control. After 24 h, 20 *μ*L of the MTT solution was added to each well. After incubation for 4 h, the medium was removed, and 100 *μ*L of DMSO was added to each well; then, absorbance (A) was detected at 490 nm using a microplate reader. The inhibition of cell growth was calculated according to the following formula: % Inhibition = [1− (A_sample_ − A_blank_) / (A_solvent_ − A_blank_)] × 100.

### 2.8 Analysis of TNF-α and IL-6 production

RAW264.7 macrophages were pretreated with 3 and 4 for 1 h prior to the stimulation with LPS (0.1 *μ*g/mL) for 6 h. The concentrations of TNF*-α* and IL-6 in the culture medium were determined using commercial ELISA kits, according to the instructions of each manufacturer.

## 3 Results and discussion

**FIGURE 2:**
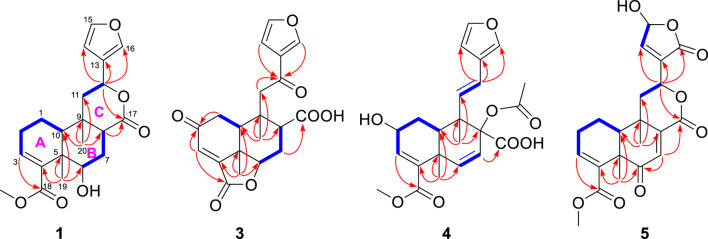
Selected ^1^H^−1^H COSY (‐) and HMBC (→) correlations of 1, 3, 4, and 5.

**FIGURE 3:**
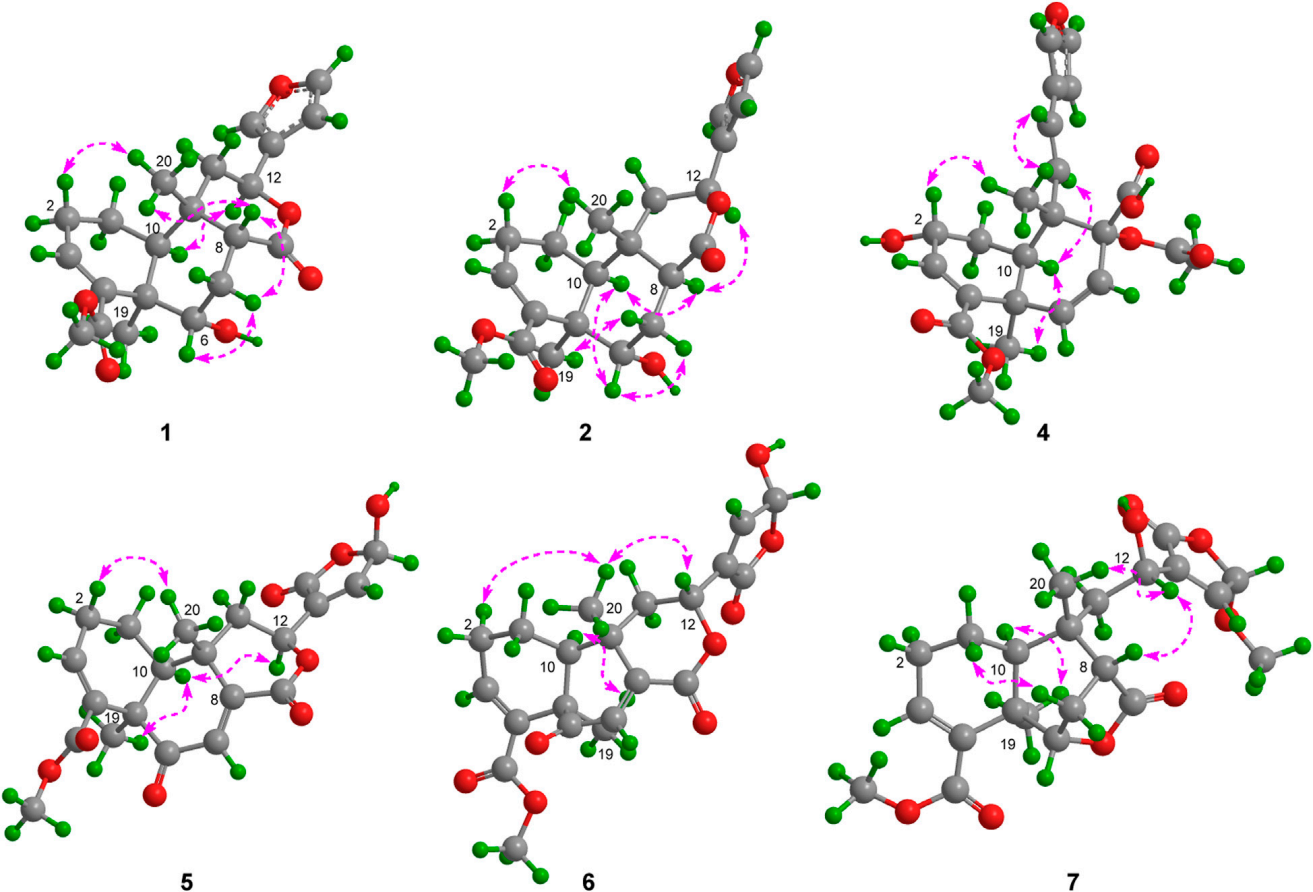
Selected NOESY correlations of metabolites 1, 2, and 4−7.

**FIGURE 4:**
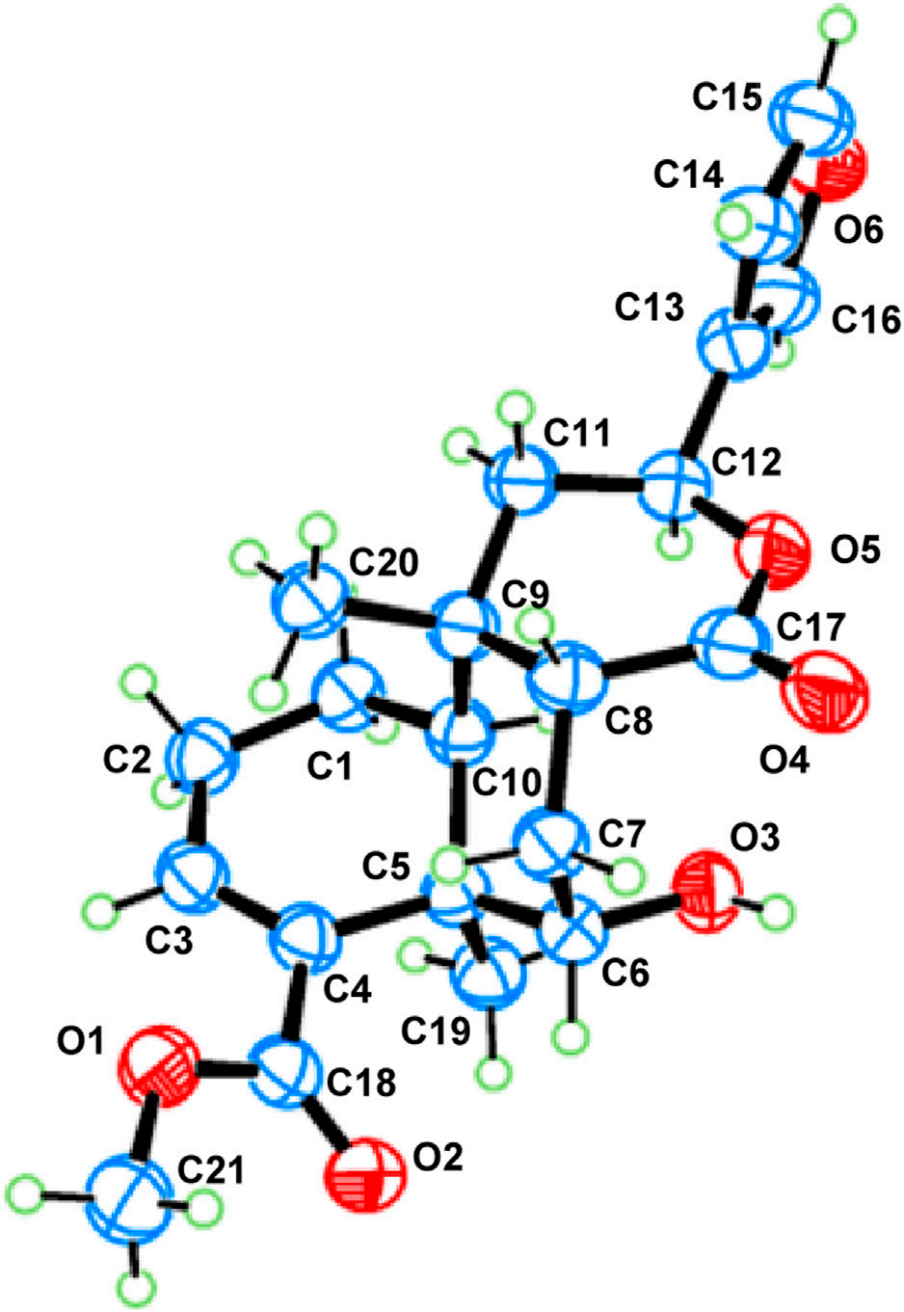
ORTEP drawing of metabolite 1.

Metabolite 1, a colorless crystal, had the molecular formula C_21_H_26_O_6_, as established by HRESIMS, which showed an ion at *m*/*z* 375.1805 [M + H]^+^ (calcd 375.1802), corresponding to nine degrees of unsaturation. The ^1^H NMR data (Table 1) displayed signals for three methyl singlets [*δ*_H_ 1.14, 1.38, and 3.74 (each 3H, s)], two oxymethine protons [*δ*_H_ 4.62 (1H, d, *J* = 5.1 Hz) and 5.55 (1H, dd, *J* = 12.6, 1.8 Hz)], a *β*-furyl ring [*δ*_H_ 7.49 (1H, brs), 7.41 (1H, brs), and 6.44 (1H, brs)], an olefinic proton [*δ*_H_ 6.77 (1H, t, *J* = 3.6 Hz)], and a series of aliphatic multiplets. The ^13^C NMR spectrum, associated with DEPT experiments, resolved 21 carbon resonances attributable to two carbonyl groups (*δ*_C_ 174.6 and 168.0), a *β*-furyl ring (*δ*_C_ 143.6, 139.7, 125.1, and 108.6), a trisubstituted double bond (*δ*_C_ 140.6 and 136.8), three methyls (one oxygenated), four sp^3^ methylenes, four sp^3^ methines (two oxygenated), and two sp^3^ quaternary carbons (Table 2). The aforementioned information was characteristic of a clerodane diterpenoid, which showed high similarity to tinopanoid R, a known clerodane recently reported from plants of the same genus (Zhu et al., 2023). Compared with tinopanoid R, the major differences in metabolite 1 were the appearance of an additional methylene (*δ*_C_ 28.8) and an additional methine (*δ*_C_ 45.7), instead of a trisubstituted double bond (*δ*_C_ 141.0 and 135.0) in tinopanoid R. This was supported by the key ^1^H–^1^H COSY correlations from H-6 (*δ*_H_ 4.62) to H-7 (*δ*_H_ 2.51 and 1.89) and H-7 to H-8 (*δ*_H_ 2.21) (Figure 2). The relative configuration of metabolite 1 was assigned mainly by the NOESY correlations (Figure 3). NOESY cross peaks between H-10/H_3_-19 and H-12/H_3_-19 and the correlation from H_3_-19 to H-7a (*δ*_H_ at 2.51) provided clear evidence for the co-facial orientation of these protons. These protons were arbitrarily assigned to *α*-orientation. Additionally, NOESY correlations from the other proton of H_2_-7 (H-7b: *δ*_H_ 1.89) to H_3_-20 and H-8 were ascertained to be in the opposite orientation. These correlations indicated that H_3_-20 and H-8 are spatially oriented in the *β*-conformation in the molecule. Finally, the absolute configuration of metabolite 1 was determined to be 5*R*, 6*S*, 8*R*, 9*S*, 10*S*, 12*S* by single-crystal X-ray diffraction analysis (Figure 4). Metabolite 1 was thus established as depicted and given the trivial name tinotanoid I, following tinotanoids A–H, which were reported from the same species.

**FIGURE 5:**
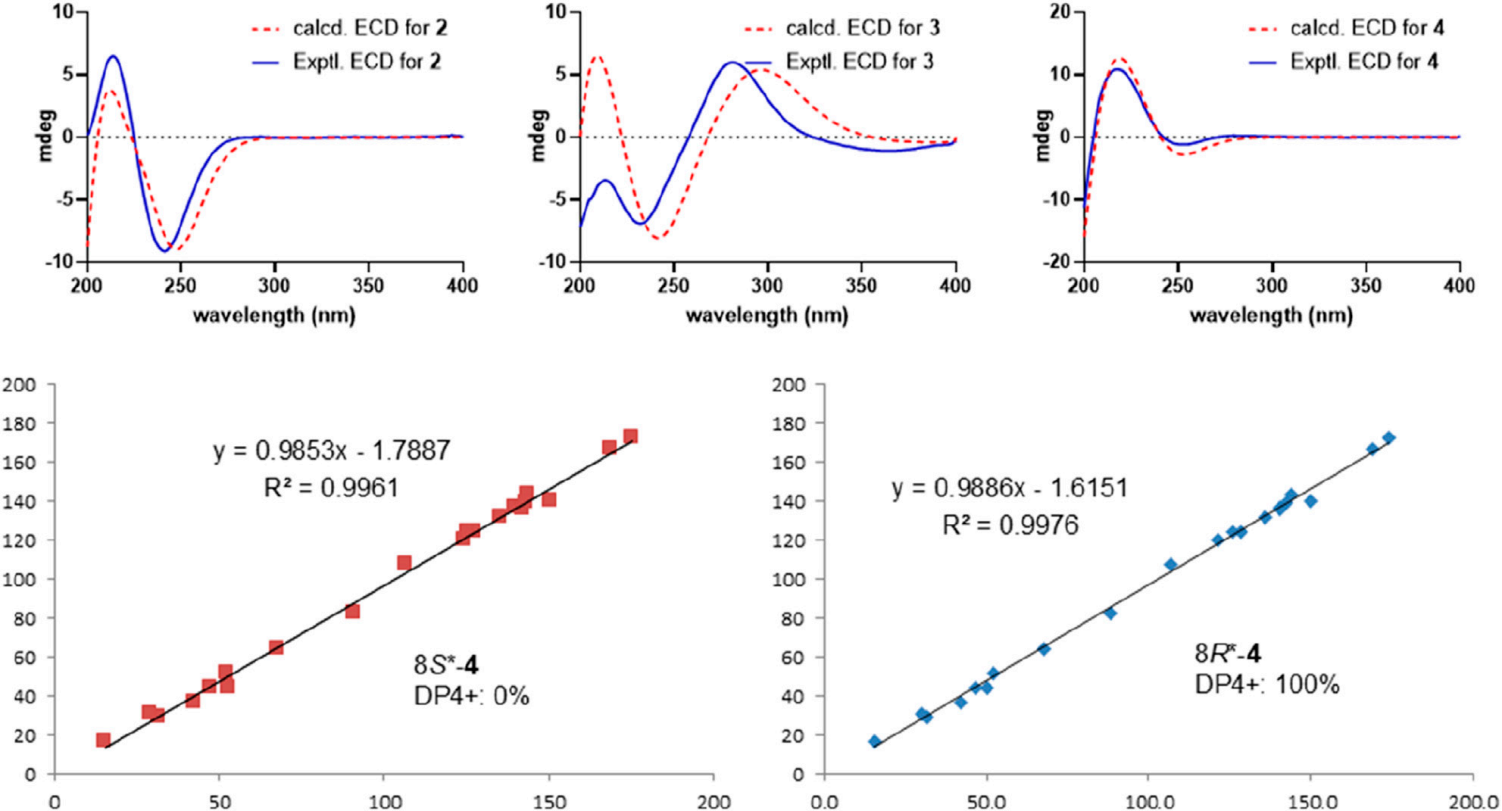
Experimental and calculated ECD spectra for metabolites 2−4 (above); NMR calculations with DP4+ probability analysis for metabolite 4 (below).

Metabolite 2, a colorless gum, had the molecular formula C_21_H_26_O_6_, as determined by the HR-ESIMS and ^13^C NMR data (Table 2). The 1D NMR spectra of metabolite 2 exhibited most of the structural features found in metabolite 1, with the major difference occurring in the signals around C-8. A detailed interpretation of 2D NMR data revealed that metabolite 2 shared the same planar structure as metabolite 1. NOESY correlations from H-10 to H-8 and H_3_-19 revealed that H-8 was in the same orientation as H-10 and H_3_-19 and was in the *α*-orientation. Thus, metabolite 2 was supposed to be the 8-epimer of 1, which was also supported by the different coupling constants of H-8 (*J* = 5.6, 4.4 Hz in 1; *J* = 12.1, 2.7 Hz in 2). The absolute configuration of metabolite 2 was assigned as 5*R*, 6*S*, 8*S*, 9*S*, 10*S*, 12*S* by ECD calculation (Figure 5). Metabolite 2 was given the trivial name tinotanoid J.

According to the HRESIMS ion peak at *m*/*z* 373.1286 [M + H]^+^ (calcd for C_20_H_21_O_7_^+^, 373.1282) and ^13^C NMR data, the molecular formula of tinotanoid K (3) was determined as C_20_H_20_O_7_ with 11 indices of hydrogen deficiency. The 1D NMR data (Tables 1, 2) suggested that metabolite 3 is a clerodane diterpenoid, exhibiting a high degree of similarity to the NMR characteristics of tinocapill B (Song Y.-Z. et al., 2024). Compared with the known tinocapill B, it was observed that metabolite 3 features a ketone group in place of the methylene unit present in the known metabolite. Key HMBC correlations from H-1 (*δ*_H_ 2.93 and 2.69), H-3 (*δ*_H_ 6.66), and H-10 (*δ*_H_ 2.65) to the additional ketone group (*δ*_C_ 197.3) revealed that the ketone group was at the C-2 position. The planar structure of metabolite 3 was further secured by a detailed interpretation of its 2D NMR data. The relative configuration of metabolite 3 was assigned to be the same as that of tinocapill B by comparing their 1D NMR data and analyzing its NOESY data. The absolute configuration of metabolite 3 was assigned as 5*R*, 6*R*, 8*R*, 9*S*, 10*S* by ECD calculation (Figure 5).

Metabolite 4, a colorless gum, had the molecular formula C_23_H_26_O_8_, as established by HRESIMS, which showed an ion at *m*/*z* 429.1551 [M − H]^−^ (calcd 429.1555), corresponding to 11 degrees of unsaturation. The 1D NMR data (Tables 1, 2) and HSQC spectrum showed signals for a *β*-furyl ring group [*δ*_H_ 7.40 (1H, brs), 7.35 (1H, brs), and 6.56 (1H, brs); *δ*_C_ 143.3, 139.6, 124.4, and 107.8], two disubstituted double bonds [*δ*_H_ 6.49 (1H, d, *J* = 10.3 Hz), 6.32 (1H, d, *J* = 10.3 Hz), 6.22 (1H, d, *J* = 16.3 Hz), and 6.14 (1H, d, *J* = 16.3 Hz); *δ*_C_ 136.1, 132.2, 124.4, and 120.0], a trisubstituted double bond [*δ*_H_ 6.70 (1H, brs); *δ*_C_ 140.1 and 136.9], two carbonyl groups [*δ*_C_ 172.5 and 166.9], an acetoxy group [*δ*_H_ 2.07 (3H, s); *δ*_C_ 169.7 and 21.0], a methoxy group [*δ*_H_ 3.74 (3H, s); *δ*_C_ 51.8], and two methyl groups [*δ*_H_ 1.54 and 0.99 (each 3H, s); *δ*_C_ 30.8 and 16.9]. The aforementioned data of metabolite 4 exhibited most of the structural features of a clerodane diterpenoid and were quite similar to those of crispene F (Al et al., 2018). Compared with crispene F, the major differences in crispene metabolite 4 were due to the appearance of an additional trans-disubstituted double bond [*δ*_H_ 6.22 (1H, d, *J* = 16.3 Hz) and 6.14 (1H, d, *J* = 16.3 Hz)] instead of one methylene and an oxymethine in crispene F. NMR comparison of the two metabolites revealed that the most signals in rings A and B were quite similar. Key HMBC correlations from the proton in the additional trans-disubstituted double bond [*δ*_H_ 6.22 and 6.14] to C-10 and C-13 suggested that the double bond was at the C-11/C-12 position. Moreover, the chemical shift at C-8 (*δ*_C_ 82.5) was downfield-shifted compared to that (*δ*_C_ 78.4) in crispene F; the absence of HMBC correlations from the oxymethine to the acetoxy group indicated that 8-OH in crispene F was acetylated. A detailed 2D NMR correlations further confirmed the planar structure of metabolite 4. The relative configuration of metabolite 4 was assigned the same as crispene F by 1D NMR and NOESY data. In particular, the stereochemistry in C-8 was not determined; the authors later established this configuration via NMR calculations and analyzed it using the DP4+ probability method. As shown in Figure 5, the final DP4+ score of 8R* (100.0%) allowed the assignment of the relative configurations of C-8. Finally, the absolute configuration of metabolite 4 was assigned by TD-DFT ECD calculation. The experimental ECD curve exhibited comparable cotton effects to the calculated ECD spectrum of (2*R*, 5*R*, 8*R*, 9*S*, 10*R*)-isomer of metabolite 4. Thus, the structure of metabolite 4 was confidently assigned as indicated and named tinotanoid L.

Metabolite 5, a colorless oil, had the molecular formula C_21_H_22_O_8_, as determined by ^13^C NMR and HRESIMS data. The ^1^H and ^13^C NMR data (Tables 1, 2) of metabolite 5 showed a ketone group (*δ*_C_ 200.2), two carbonyl groups [*δ*_C_ 167.0 and 166.5], a *β*-butenolide moiety [*δ*_H_ 7.31 (1H, brs) and 6.22 (1H, brs); *δ*_C_ 168.5, 145.9, 135.0, and 97.6], two trisubstituted double bonds [*δ*_H_ 6.78 (1H, t, *J* = 4.0 Hz) and 6.65 (1H, s); *δ*_C_ 148.2, 139.0, 134.2, and 131.0], a methoxy group [*δ*_H_ 3.70 (3H, s); *δ*_C_ 52.1], and two methyl groups [*δ*_H_ 1.44 and 1.26 (each 3H, s); *δ*_C_ 26.2 and 26.1]. The abovementioned data were quite similar to those of tinocordifoliol A (Hoang et al., 2024), with notable differences being the absence of the *β*-furyl ring in tinocordifoliol A and the presence of a *β*-butenolide moiety in metabolite 5. HMBC correlations from H-12 (*δ*_H_ 4.93) to the carbons of the *β*-butenolide moiety (*δ*_C_ 168.5, 145.9, and 135.0) supported the above elucidation. 2D NMR analysis established the molecular structure of metabolite 5. The relative configuration of metabolite 5 was determined by 1D NMR data, compared to the known metabolite, along with the NOESY spectrum (Figure 3). Key NOESY correlations from H_3_-19 to H-10 assigned these protons as having *α*-orientation, similar to those of tinocordifoliol A. Thus, NOESY correlations from H-12 to H-10 revealed that H-12 and H-10 were co-facial and in the *α*-orientation. Thus, metabolite 5 was named tinotanoid M.

HRESIMS analysis revealed that the molecular ion at *m*/*z* 401.1243 of tinotanoid N (6) was consistent with the molecular formula C_21_H_22_O_8_ (calcd 401.1242), indicating that it was an isomer of metabolite 5. Comprehensive analyses of the NMR spectrum revealed that metabolite 6 shared the same planar structure as metabolite 5. The configuration of H-10/H_3_-19 in the *α*-orientation, along with H_3_-20 in the *β*-orientation, was assigned as the same as in metabolite 5, according to the biosynthesis consideration. A comprehensive analysis of NOESY correlations revealed the signals of H-12/H_3_-20, indicating that H-12 was in the *β*-orientation. Thus, metabolite 6 was assigned as 12-isomer of metabolite 5.

Metabolites 5 and 6 are both expected to be racemates at the C-15 position as a result of the hemiacetal functionality, leading to dynamic interconversion at this site. Nonetheless, spectral data indicate a high degree of purity for both metabolites, potentially due to hydrogen bonding interactions. The exact mechanism behind this phenomenon is a subject for further investigation. The absolute configurations of 5 and 6 were determined by ECD calculation (see Supplementary Figure S50).

Metabolite 7 had the molecular formula C_22_H_24_O_8_. The NMR spectra of metabolite 7 showed the signals of a *β*-butenolide moiety [*δ*_H_ 7.03 (1H, s) and 5.78 (1H, s); *δ*_C_ 170.7, 142.2, 140.0, and 103.0], two carbonyl groups [*δ*_C_ 179.1 and 166.7], a trisubstituted double bond [*δ*_H_ 7.02 (1H, brt, *J* = 3.5 Hz); *δ*_C_ 142.6 and 134.1], two methoxy groups [*δ*_H_ 3.72 and 3.56 (each 3H, s); *δ*_C_ 57.2 and 51.7], and two methyl groups [*δ*_H_ 1.34 and 1.22 (each 3H, s); *δ*_C_ 27.1 and 21.8] (Tables 1, 2). The abovementioned data were quite similar to those of tinocapill C, except for the absence of the ethyl unit, indicating that metabolite 7 was the deethylated product of tinocapill C (Song J.-Q. et al., 2024). A detailed 2D NMR analysis revealed the planar structure of metabolite 7. The relative configuration of metabolite 7 was assigned the same as that of tinocapill C by their highly matched 1D NMR data and NOESY correlations. In particular, NOESY correlations from H-12 to H-8 and H_3_-20, along with the similar 1D NMR data of tinocapill C and metabolite 7 [*δ*_H_ 4.92 and *δ*_C_ 64.4 in tinocapill C; *δ*_H_ 4.89 and *δ*_C_ 64.8 in metabolite 7], indicated that H-12 was *β*-oriented. Thus, metabolite 7 was named tinotanoid O.

Metabolites 1−7 were assessed for their ability to inhibit NO production in LPS-stimulated RAW264.7 macrophages. Quercetin, a recognized natural inhibitor of NO, and dexamethasone, a broad-spectrum anti-inflammatory drug, were employed as positive controls with IC_50_ values of 21.0 ± 0.6 and 24.5 ± 1.9 *μ*M, respectively. The results presented in Table 3 indicate that metabolites 3 and 4 demonstrated significant inhibitory effects, with IC_50_ values of 12.5 ± 0.5 and 16.4 ± 0.7 *μ*M, respectively. In contrast, the remaining metabolites, with IC_50_ values exceeding 50 *μ*M, were deemed inactive. To rule out the possibility that the inhibitory effects were due to cytotoxicity, the viability of RAW264.7 cells treated with the active metabolites was evaluated using the MTT assay. No appreciable cytotoxicity was noted, with cell survival rates exceeding 90% at all the concentrations below 50 *μ*M, suggesting that the inhibitory activities observed were not a result of cellular toxicity.

**TABLE 3:** IC_50_ values of the active metabolites on LPS-induced NO production in RAW264.7 cells.

No.	IC_50_ (*μ*M)	No.	IC_50_ (*μ*M)
1	>50	2	>50
3	12.5 ± 0.5	4	16.4 ± 0.7
5	>50	6	>50
7	>50	Quercetin^*a*^	21.0 ± 0.6
Dexamethasone^*a*^	24.5 ± 1.9	^*a*^positive control	

**FIGURE 6:**
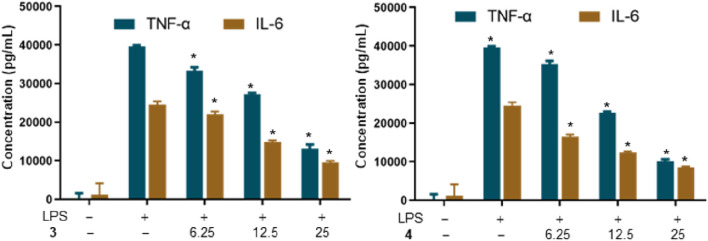
Effects of metabolites 3 and 4 on the production of TNF-*α* in LPS-induced RAW264.7 macrophages (*p < 0.05 versus the group treated with LPS only).

An overabundance of pro-inflammatory mediators, including TNF-*α*, IL-1, IL-6, NO, and PGE2, secreted by macrophages, can precipitate pathological conditions and is associated with various chronic inflammatory diseases (Chan et al., 2015). Notably, TNF-*α* and IL-6 are considered key biomarkers for evaluating the inflammatory state. To delve into the anti-inflammatory capabilities of metabolites 3 and 4, their ability to inhibit LPS-induced expression of TNF-*α* and IL-6 in RAW 264.7 cells was examined. Figure 6 reveals that the elevated levels of TNF-*α* and IL-6 following LPS stimulation were significantly reduced by pretreatment with metabolites 3 and 4 with a dose-dependent inhibition. These findings indicate that metabolites 3 and 4 have the potential to mitigate the inflammatory response in RAW264.7 cells.

## 4 Conclusion

*Tinospora sagittata*, a revered traditional Chinese medicinal botanical drug, is renowned for its potent anti-inflammatory properties. Previous studies have demonstrated that clerodane diterpenoids extracted from *T. sagittata* exhibit a range of anti-inflammatory effects (Li et al., 2012; Song Y.-Z. et al., 2024). In the current study, seven previously unreported clerodane diterpenoids were successfully isolated from the tuberous roots of *T. sagittata* var. yunnanensis, two of which showed potent anti-inflammatory activity. These findings suggest that the anti-inflammatory properties of *T*. *sagittata* may be attributed to clerodane diterpenoids. Additionally, the specific clerodane diterpenoids identified in this study could serve as promising candidates for further pharmacological investigation and may contribute to the advancement of botanical drugs in the treatment of inflammatory diseases. Furthermore, exploring the mechanisms by which these clerodane diterpenoids modulate key inflammatory pathways, such as NF-κB and MAPK, could be a promising strategy to enhance their pharmacological significance.

## Data Availability

The original contributions presented in the study are included in the article/Supplementary Material; further inquiries can be directed to the corresponding author.
