# Supplementary material for: Clerodane diterpenoids with anti-inflammatory activity from the tuberous root of Paratinospora sagittata (Oliv.) Wei Wang
Source: Front Pharmacol. 2025 Mar 20;16:1561954. doi: 10.3389/fphar.2025.1561954 (PMC11966029; doi:10.3389/fphar.2025.1561954)
Supplement: Supplementary file 1 [file DataSheet1.pdf]

Supplementary Material

Clerodane Diterpenoids with anti-inflammatory activity from the tuberous root of *Tinospora sagittate* var. yunnanensis

Contents:

S1. ^{1}H NMR Spectrum of 1 in $CDCl_3$

S2. ^{13}C NMR Spectrum of 1 in $CDCl_3$

S3. ^{1}H-^{1}H COSY Spectrum of 1 in $CDCl_3$

S4. HSQC Spectrum of 1 in $CDCl_3$

S5. HMBC Spectrum of 1 in $CDCl_3$

S6. NOESY Spectrum of 1 in $CDCl_3$

S7. HRESIMS Spectrum of 1

S8. ^{1}H NMR Spectrum of 2 in $CDCl_3$

S9. ^{13}C NMR Spectrum of 2 in $CDCl_3$

S10. ^{1}H-^{1}H COSY Spectrum of 2 in $CDCl_3$

S11. HSQC Spectrum of 2 in $CDCl_3$

S12. HMBC Spectrum of 2 in $CDCl_3$

S13. NOESY Spectrum of 2 in $CDCl_3$

S14. HRESIMS Spectrum of 2

S15. ^{1}H NMR Spectrum of 3 in $CDCl_3$

S16. ^{13}C NMR Spectrum of 3 in $CDCl_3$

S17. ^{1}H-^{1}H COSY Spectrum of 3 in $CDCl_3$

S18. HSQC Spectrum of 3 in $CDCl_3$

S19. HMBC Spectrum of 3 in $CDCl_3$

S20. NOESY Spectrum of 3 in $CDCl_3$

S21. HRESIMS Spectrum of 3

S22. ^{1}H NMR Spectrum of 4 in $CDCl_3$

S23. ^{13}C NMR Spectrum of 4 in $CDCl_3$

S24. ^{1}H-^{1}H COSY Spectrum of 4 in $CDCl_3$

S25. HSQC Spectrum of 4 in $CDCl_3$

S26. HMBC Spectrum of 4 in $CDCl_3$

S27. NOESY Spectrum of 4 in $CDCl_3$

S28. HRESIMS Spectrum of 4

S29. ^{1}H NMR Spectrum of 5 in $CDCl_3$

S30. ^{13}C NMR Spectrum of 5 in $CDCl_3$

S31. ^{1}H-^{1}H COSY Spectrum of 5 in $CDCl_3$

S32. HSQC Spectrum of 5 in $CDCl_3$

S33. HMBC Spectrum of 5 in $CDCl_3$

S34. NOESY Spectrum of 5 in $CDCl_3$

S35. HRESIMS Spectrum of 5

S36. ^{1}H NMR Spectrum of 6 in $CDCl_3$

S37. ^{13}C NMR Spectrum of 6 in $CDCl_3$

S38. ^{1}H-^{1}H COSY Spectrum of 6 in $CDCl_3$

S39. HSQC Spectrum of 6 in $CDCl_3$

S40. HMBC Spectrum of 6 in $CDCl_3$

S41. NOESY Spectrum of 6 in $CDCl_3$

S42. HRESIMS Spectrum of 6

S43. ^{1}H NMR Spectrum of 7 in $CDCl_3$

S44. ^{13}C NMR Spectrum of 7 in $CDCl_3$

S45. ^{1}H-^{1}H COSY Spectrum of 7 in $CDCl_3$

S46. HSQC Spectrum of 7 in $CDCl_3$

S47. HMBC Spectrum of 7 in $CDCl_3$

S48. NOESY Spectrum of 7 in $CDCl_3$

S49. HRESIMS Spectrum of 7

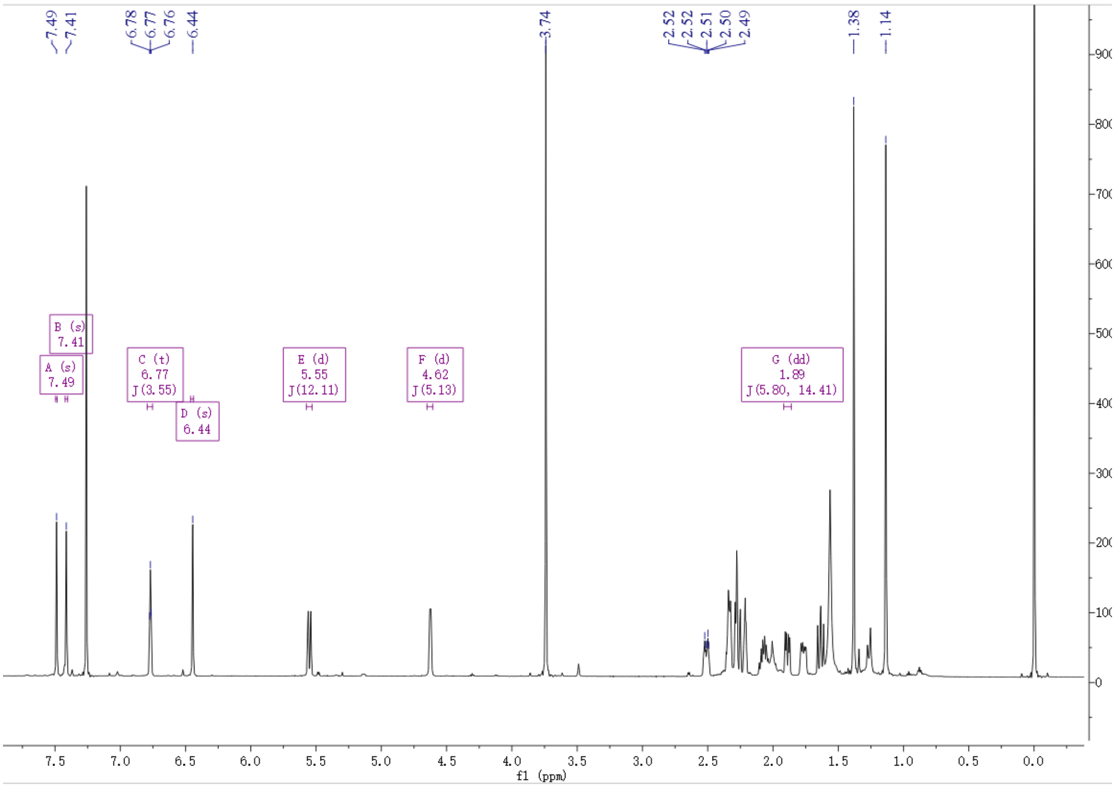

S1. ^{1}H NMR Spectrum of 1 in $CDCl_3$

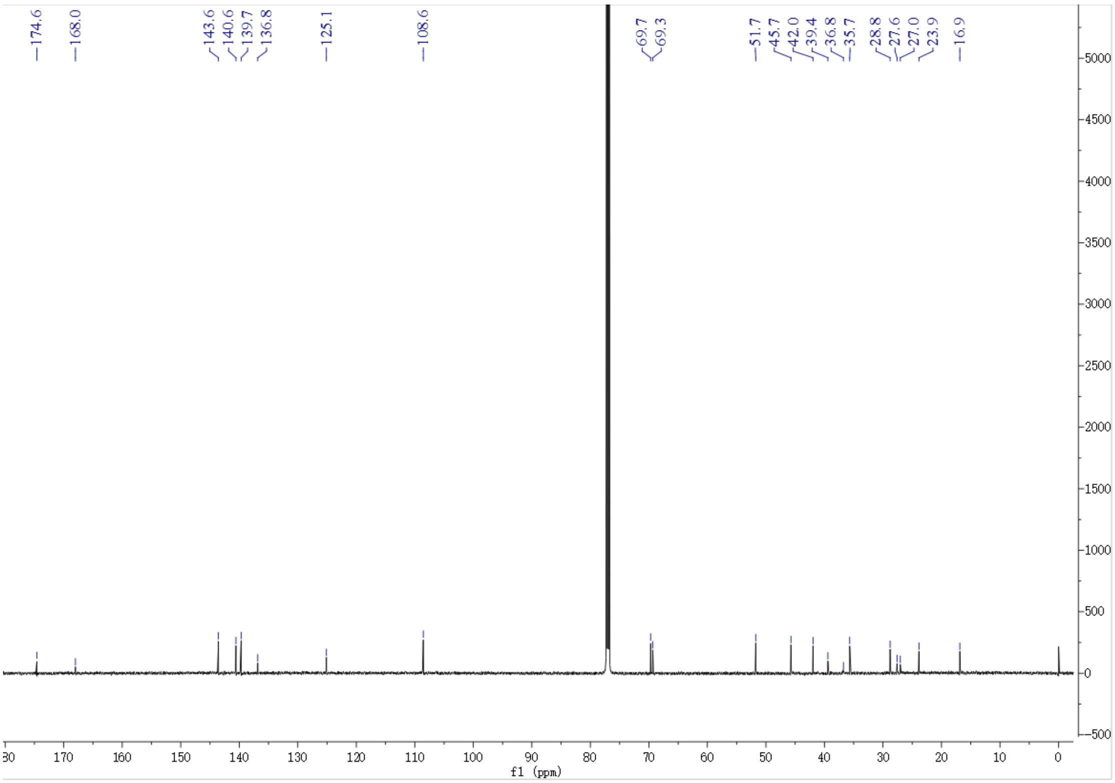

S2. ^{13}C NMR Spectrum of 1 in $CDCl_3$

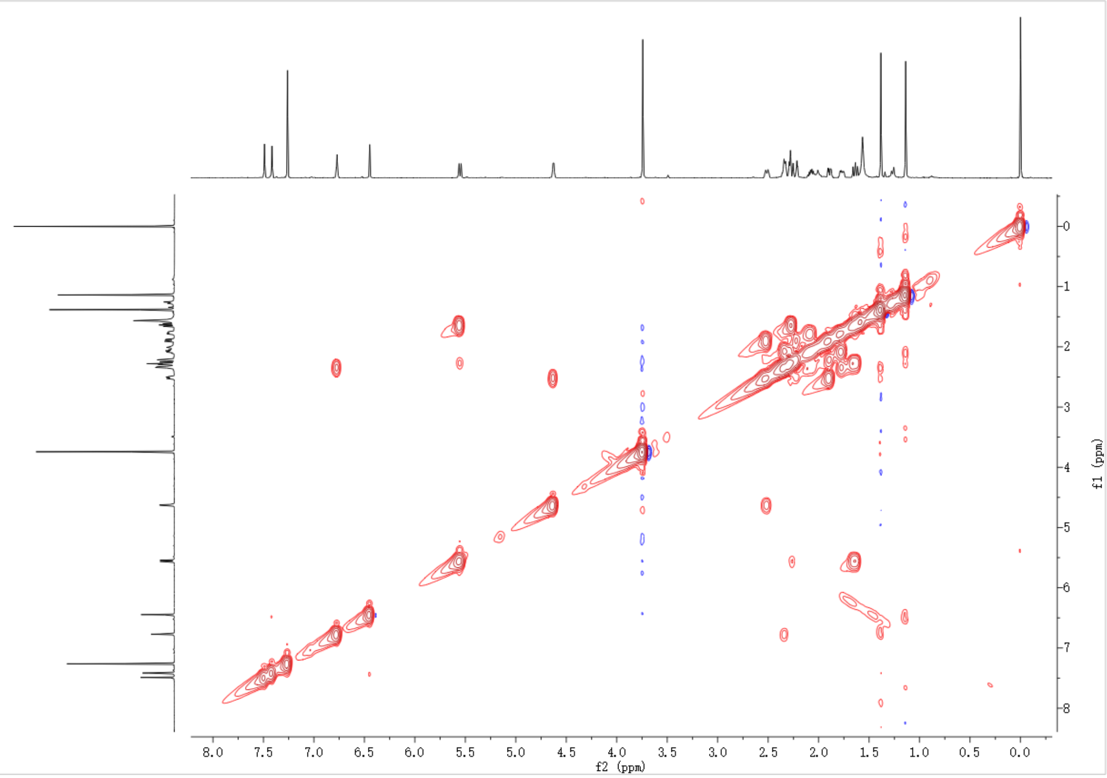

S3. ^{1}H-^{1}H COSY Spectrum of 1 in $CDCl_3$

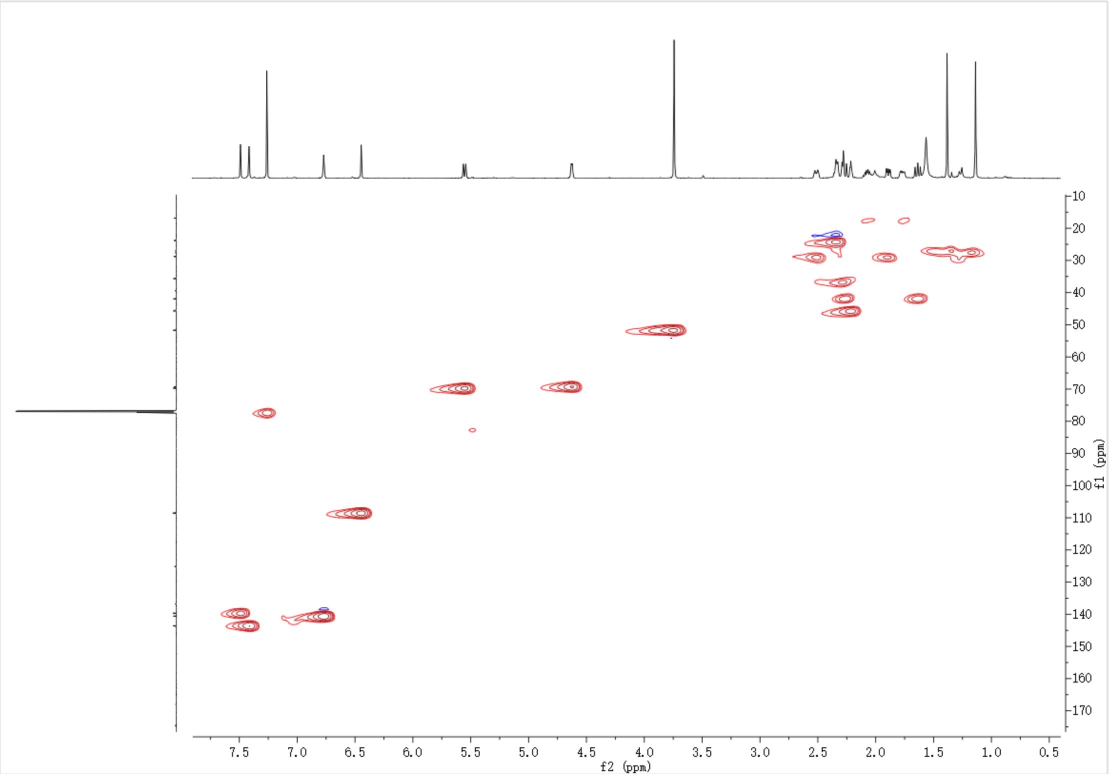

S4. HSQC Spectrum of 1 in $CDCl_3$

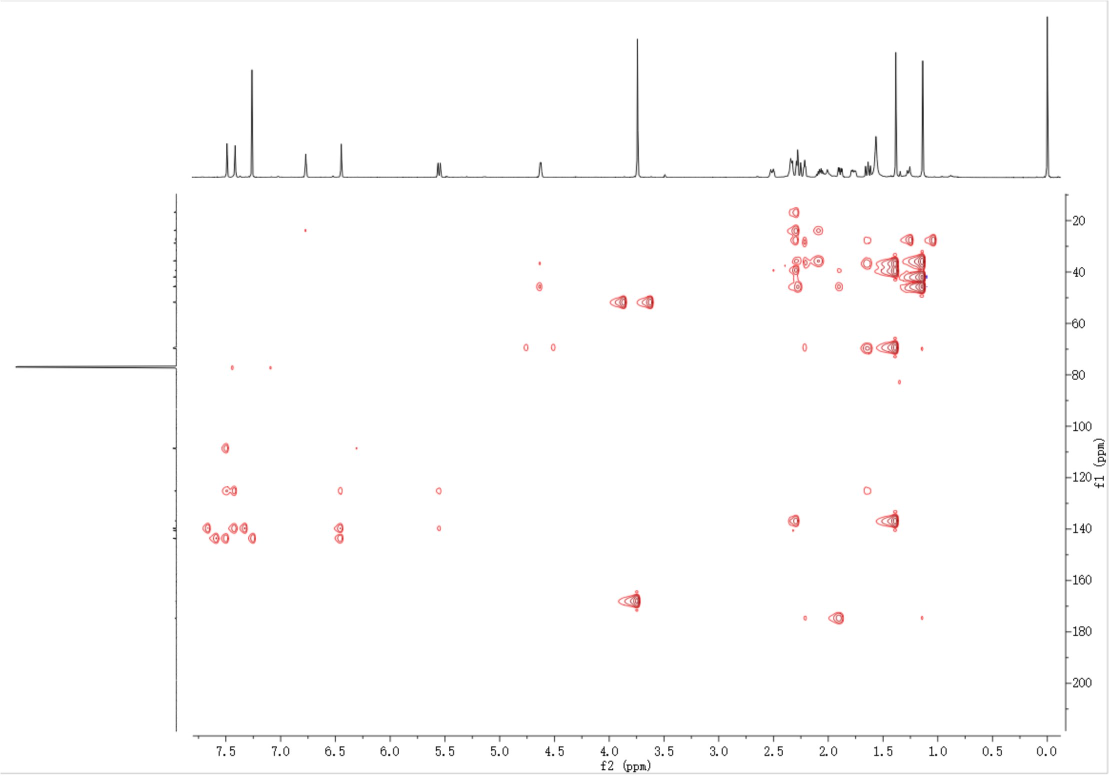

S5. HMBC Spectrum of 1 in $CDCl_3$

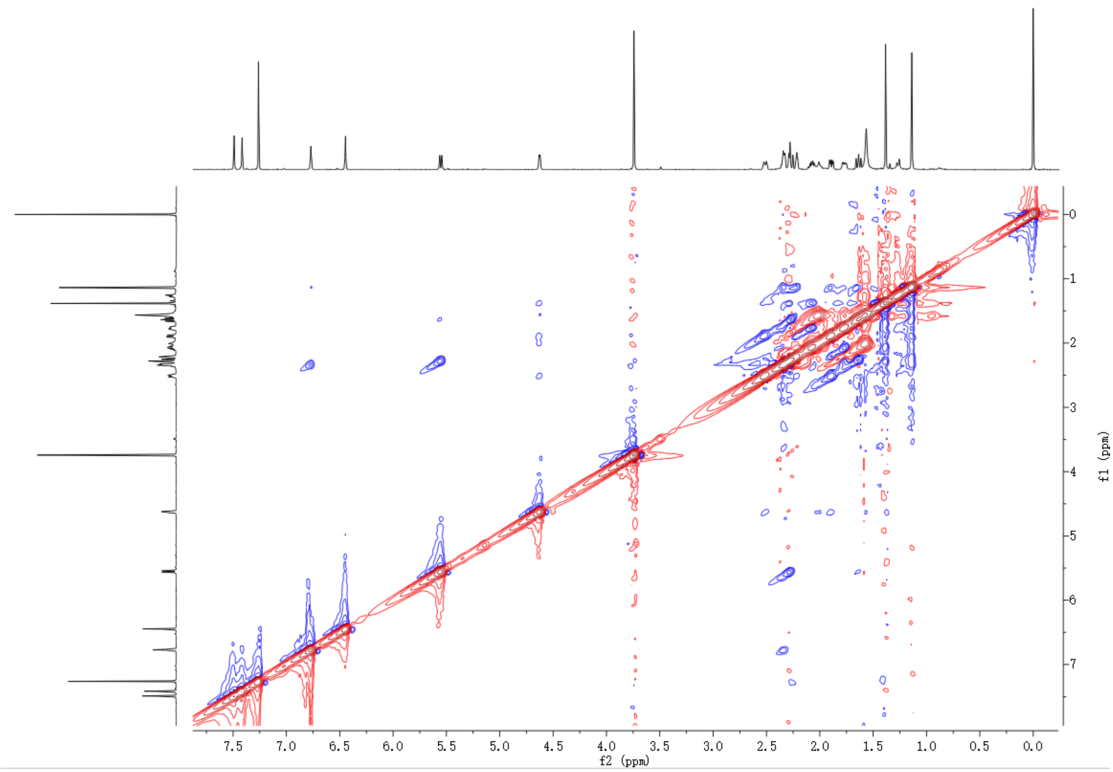

S6. NOESY Spectrum of 1 in $CDCl_3$

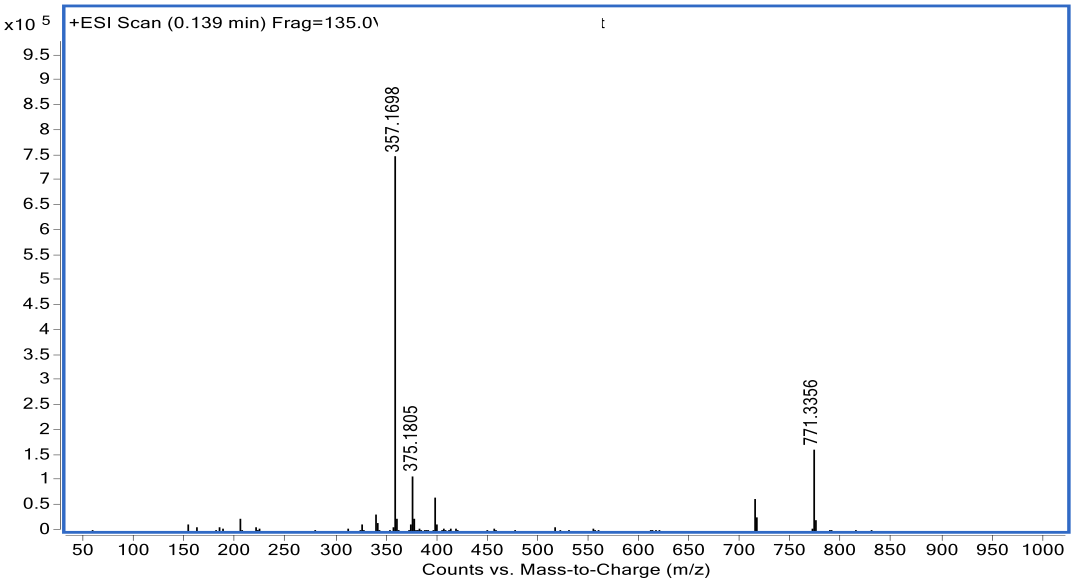

S7. HRESIMS Spectrum of 1

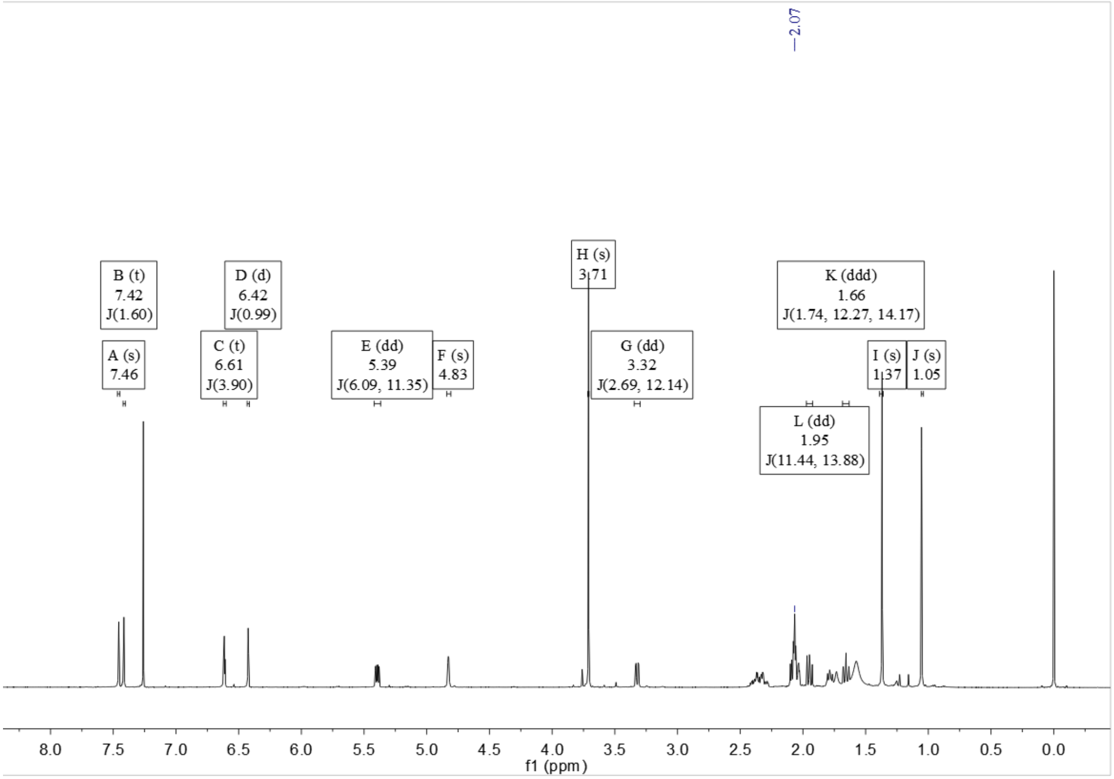

S8. ^{1}H NMR Spectrum of 2 in $CDCl_3$

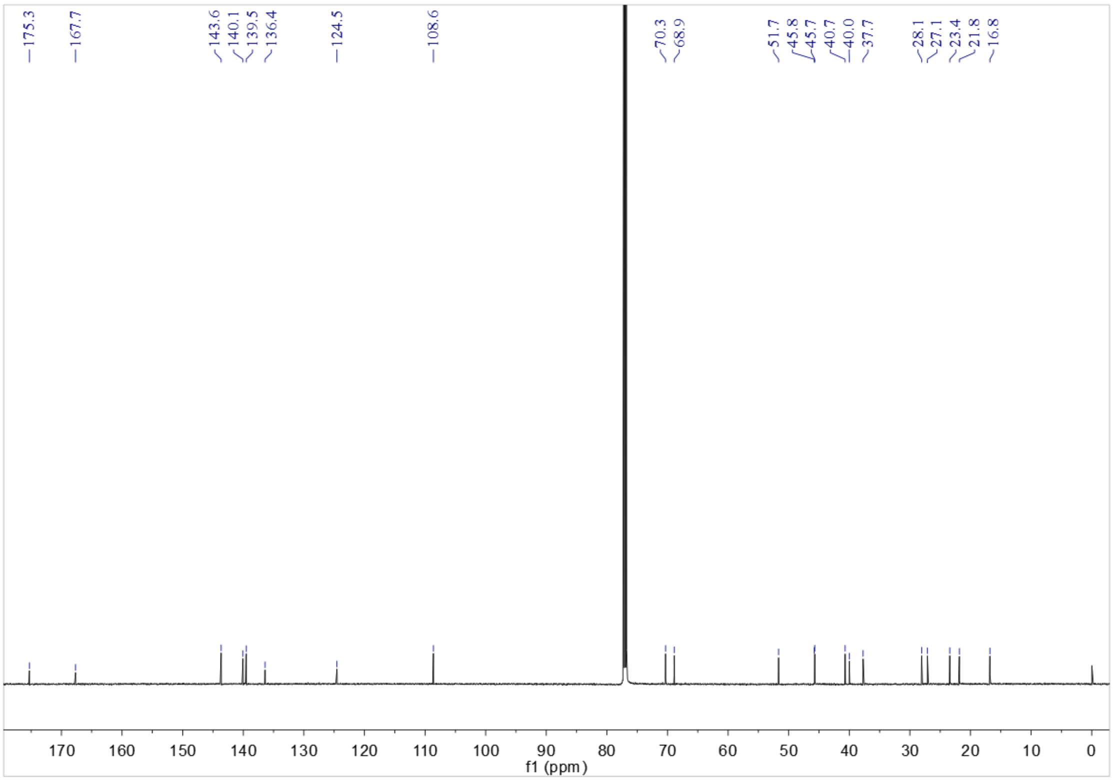

S9. ^{13}C NMR Spectrum of 2 in $CDCl_3$

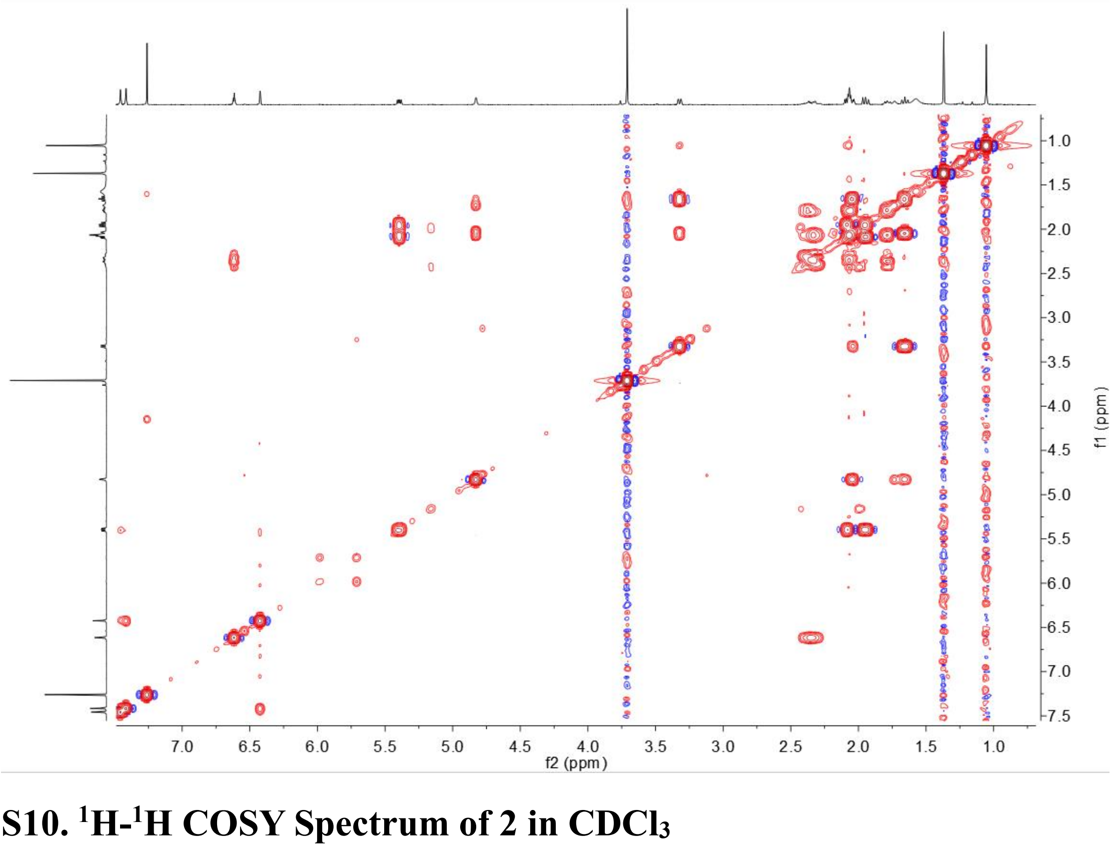

S10. ^{1}H-^{1}H COSY Spectrum of 2 in $CDCl_3$

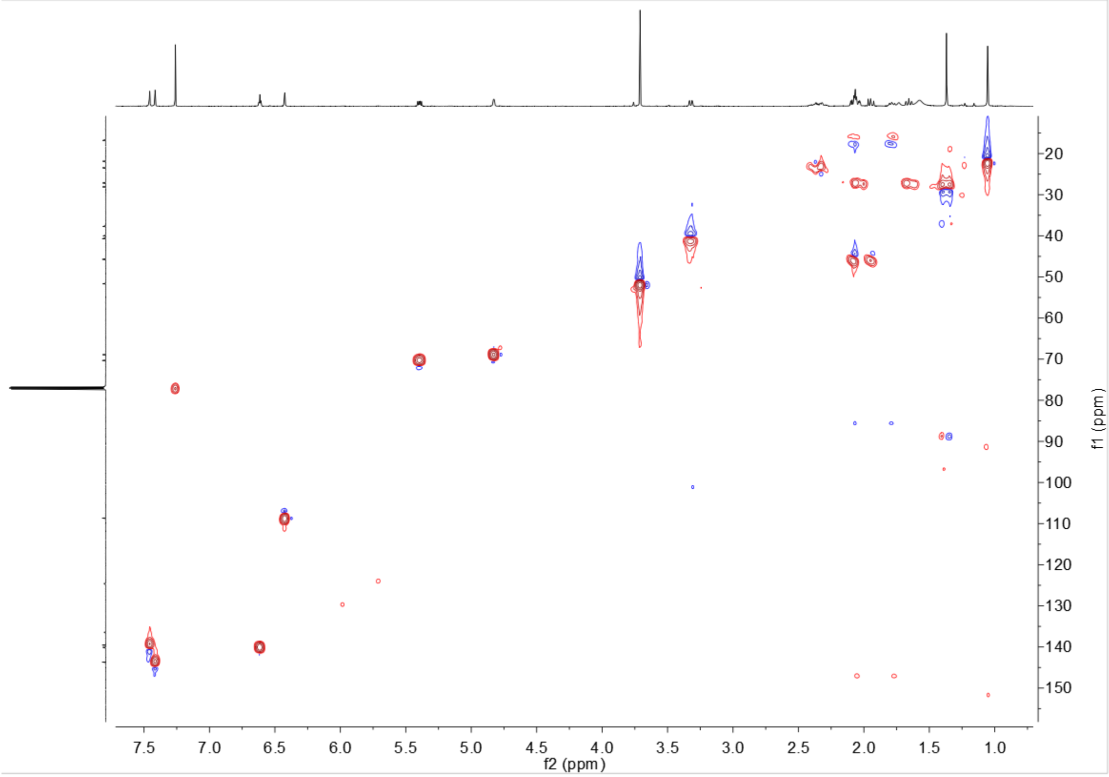

S11. HSQC Spectrum of 2 in $CDCl_3$

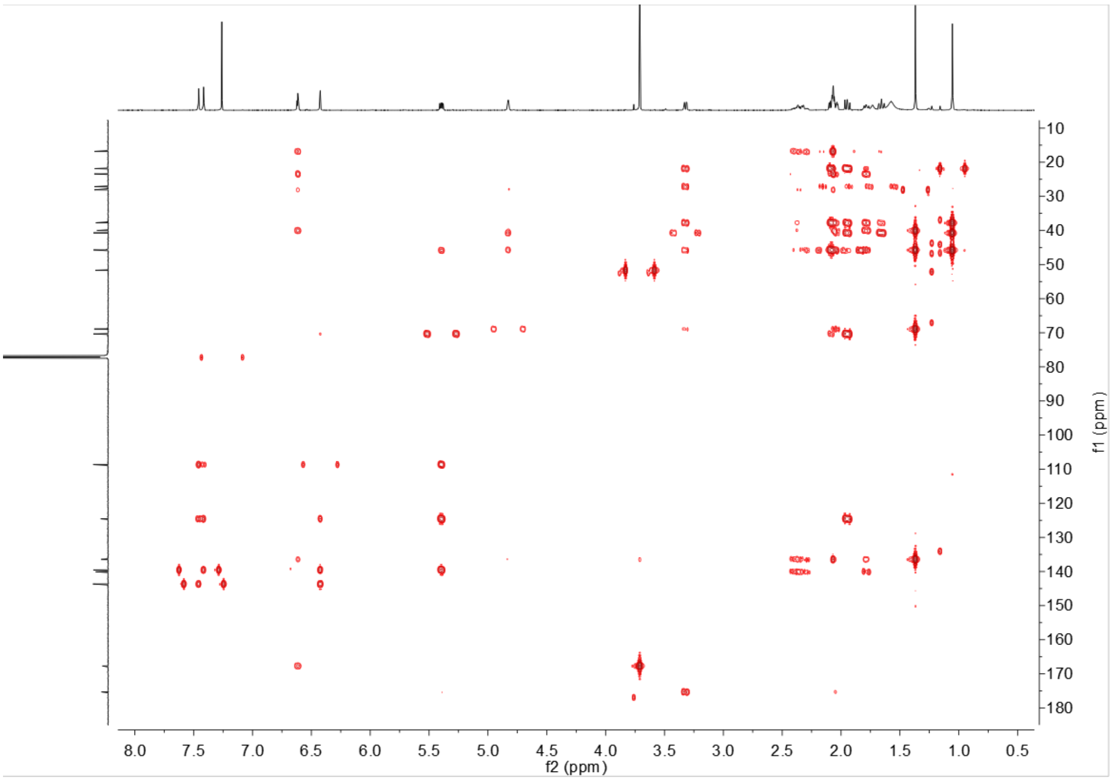

S12. HMBC Spectrum of 2 in $CDCl_3$

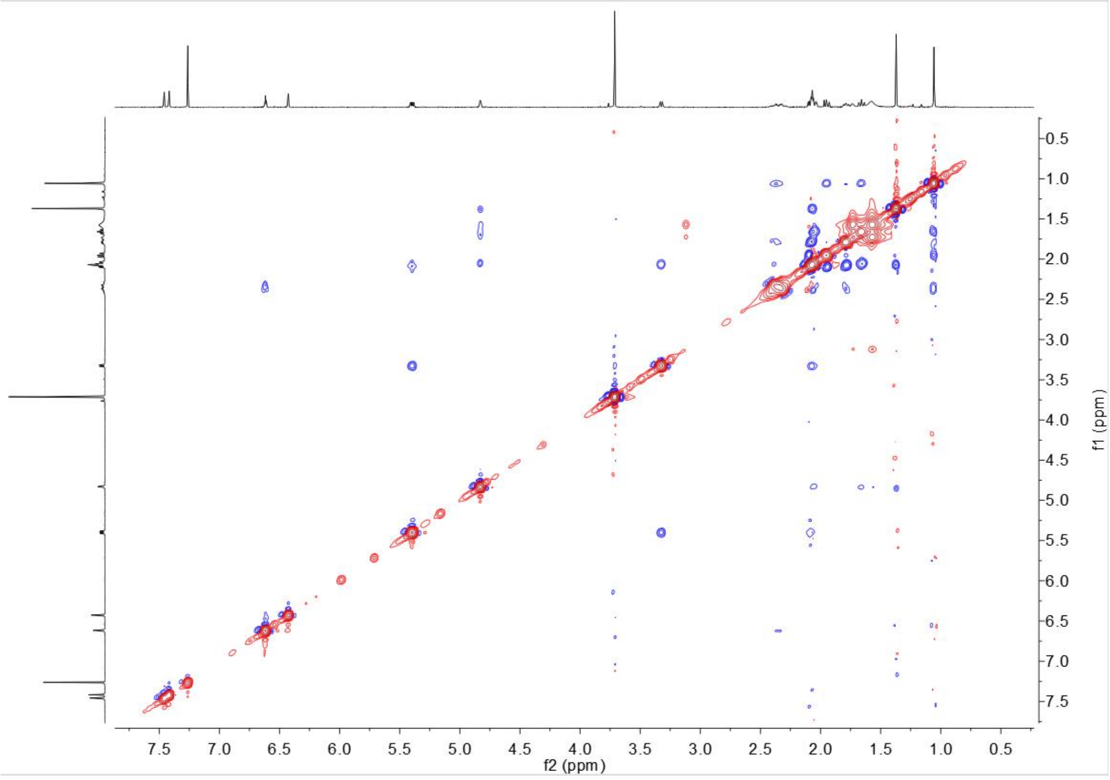

S13. NOESY Spectrum of 2 in $CDCl_3$

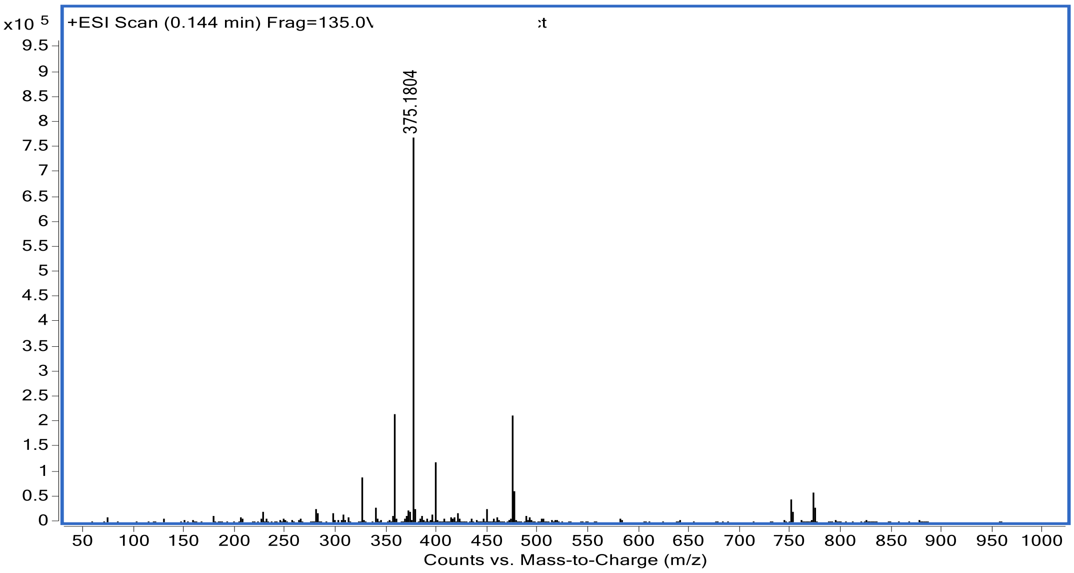

S14. HRESIMS Spectrum of 2

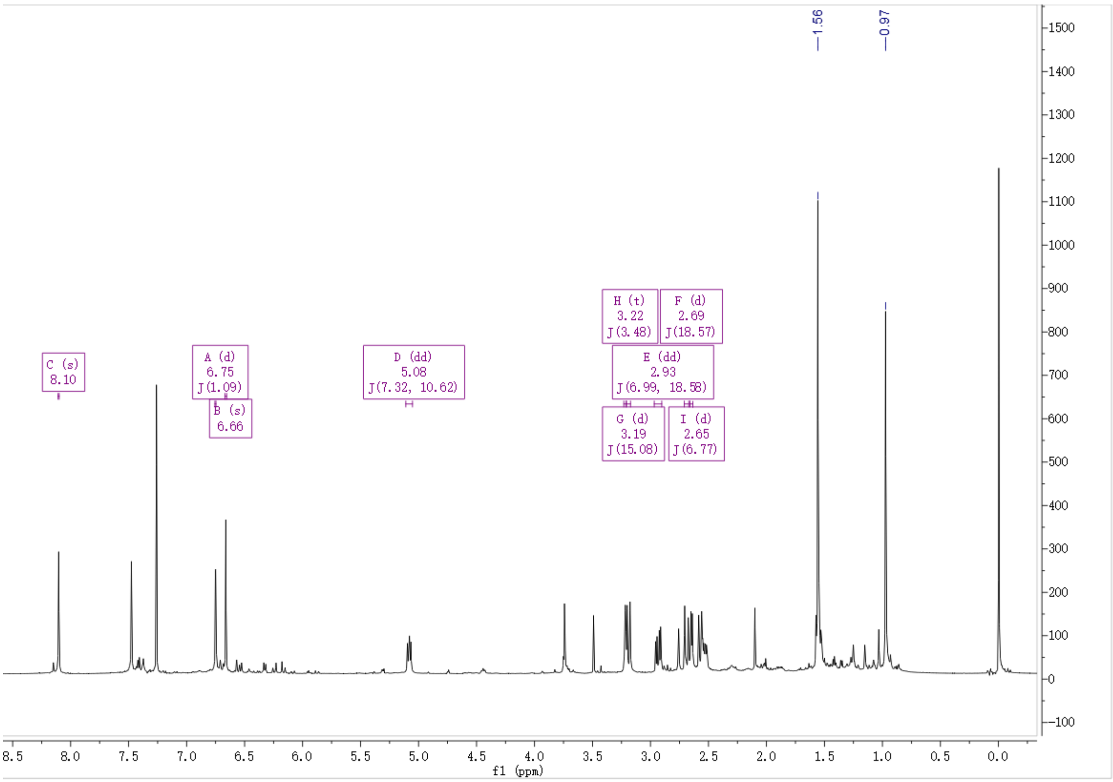

S15. ^{1}H NMR Spectrum of 3 in $CDCl_3$

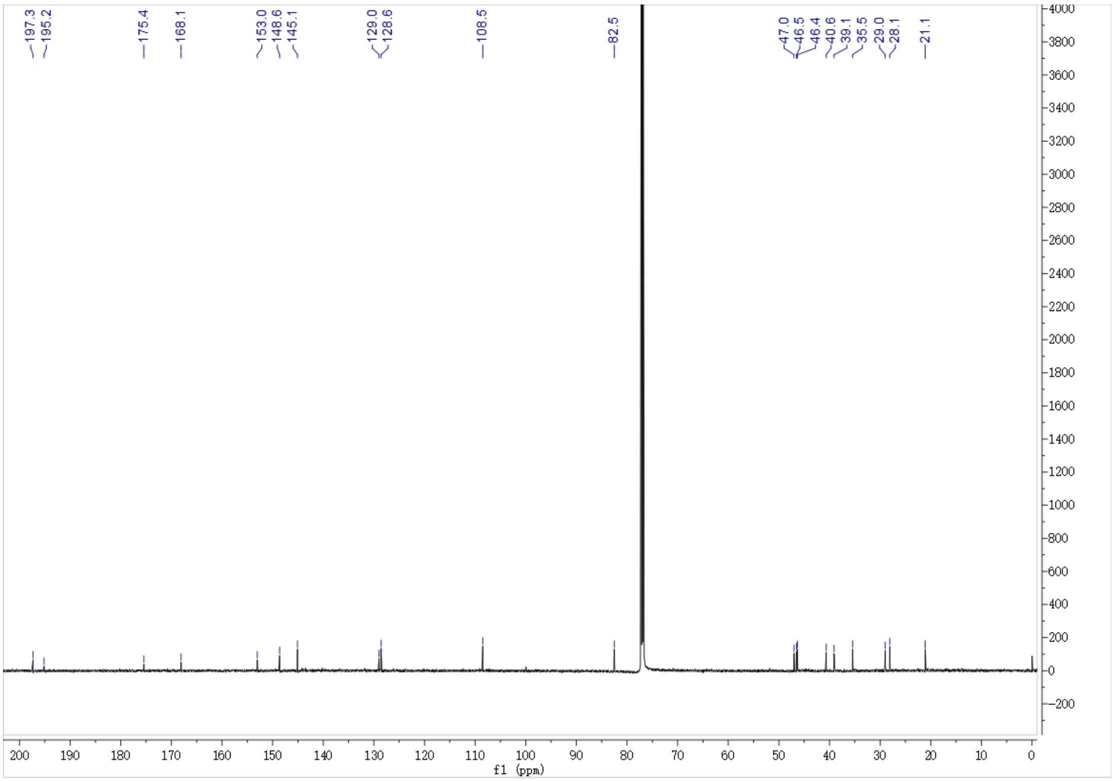

S16. ^{13}C NMR Spectrum of 3 in $CDCl_3$

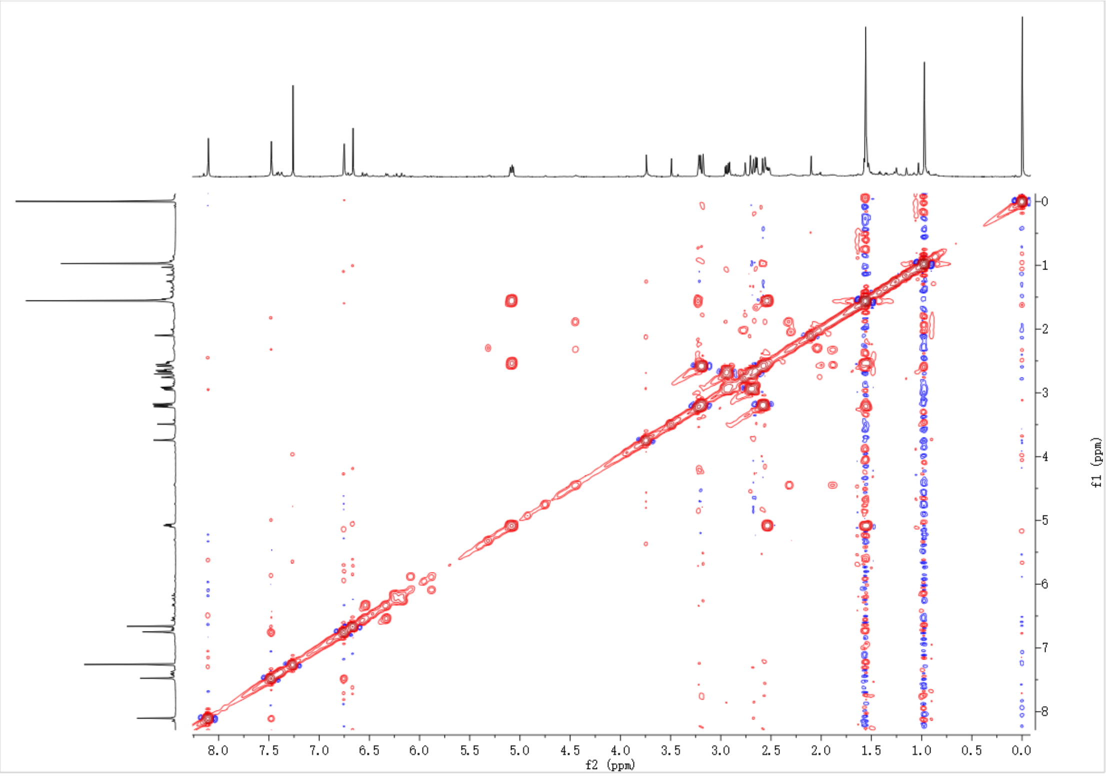

S17. ^1H-^1H COSY Spectrum of 3 in $CDCl_3$

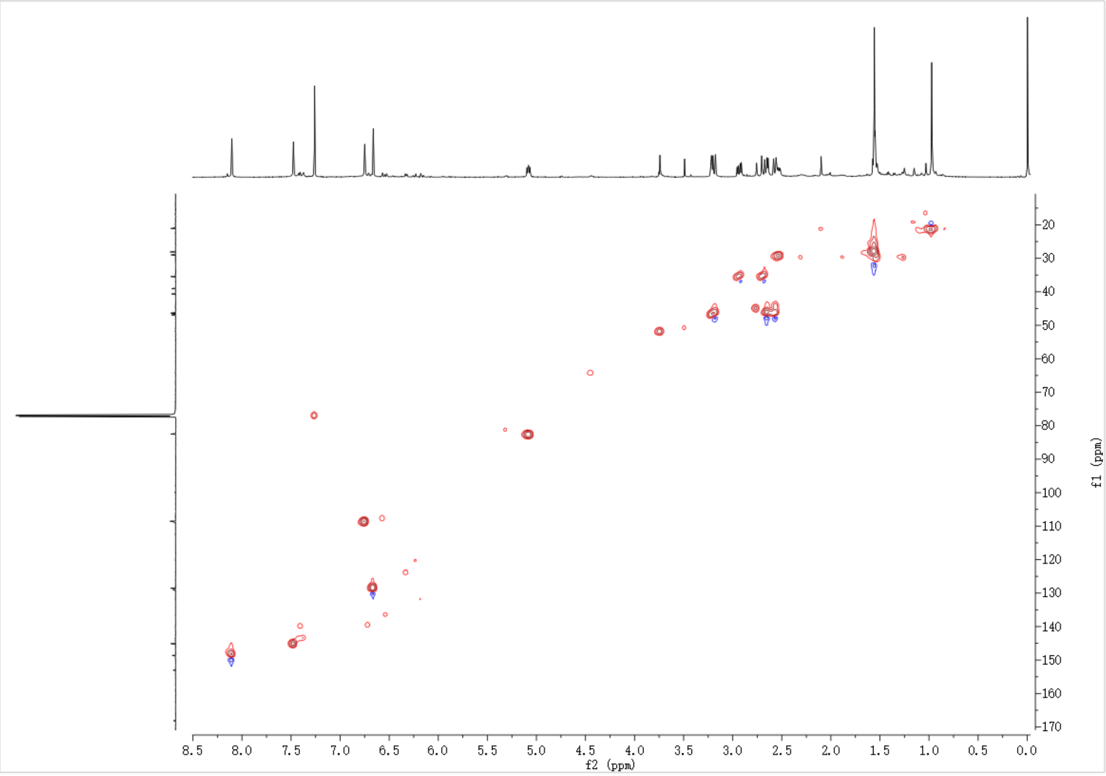

S18. HSQC Spectrum of 3 in $CDCl_3$

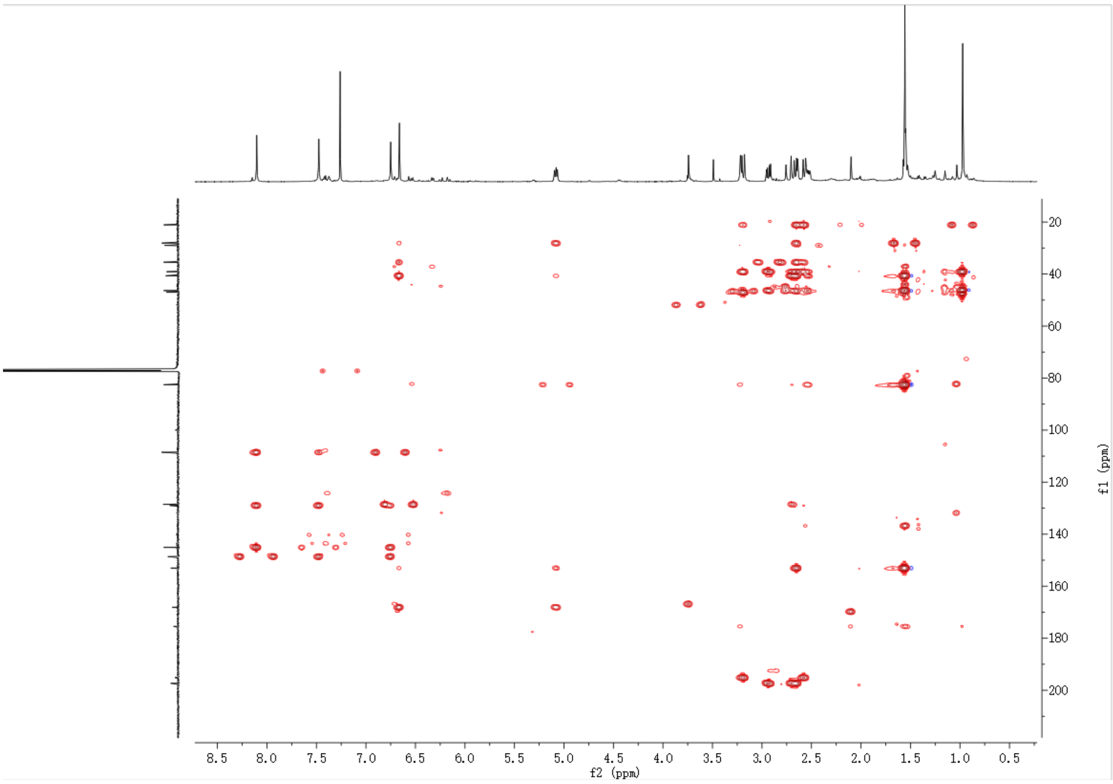

S19. HMBC Spectrum of 3 in $CDCl_3$

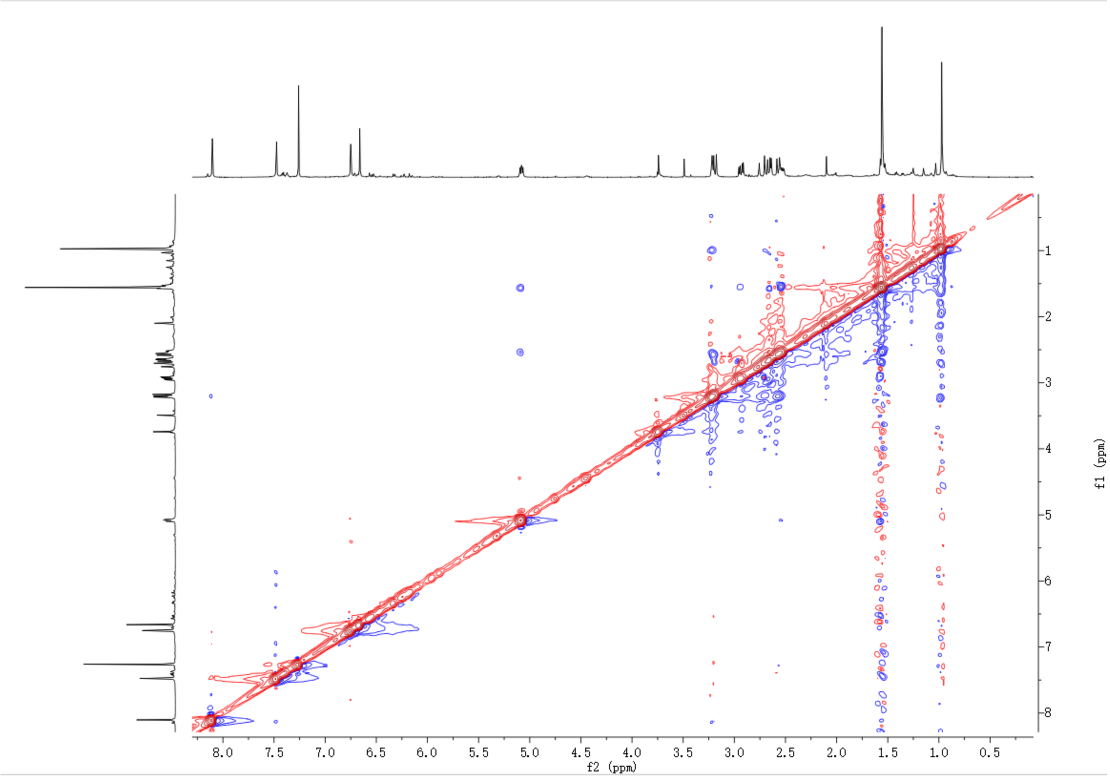

S20. NOESY Spectrum of 3 in $CDCl_3$

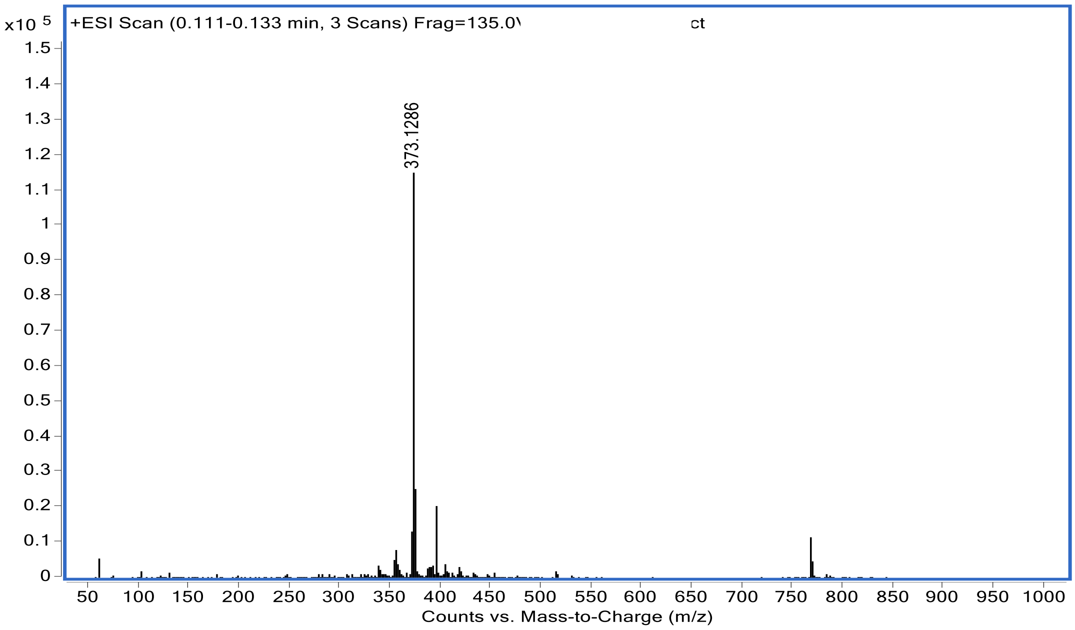

S21. HRESIMS Spectrum of 3

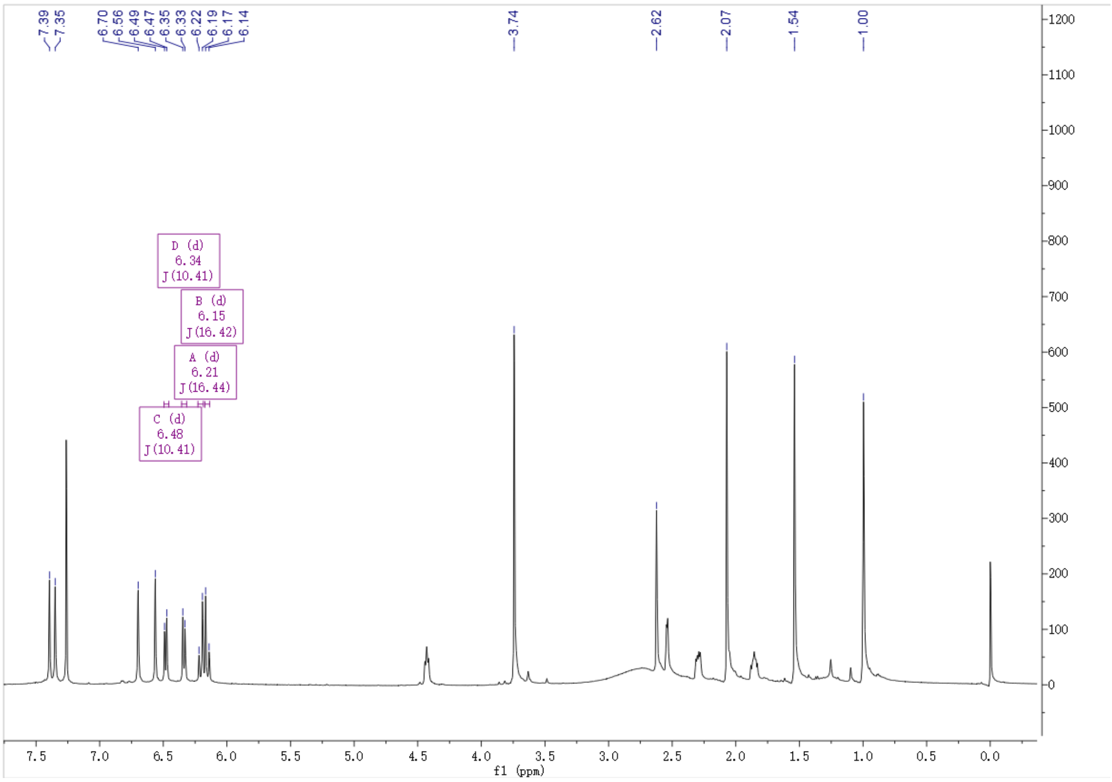

S22. ^{1}H NMR Spectrum of 4 in $CDCl_3$

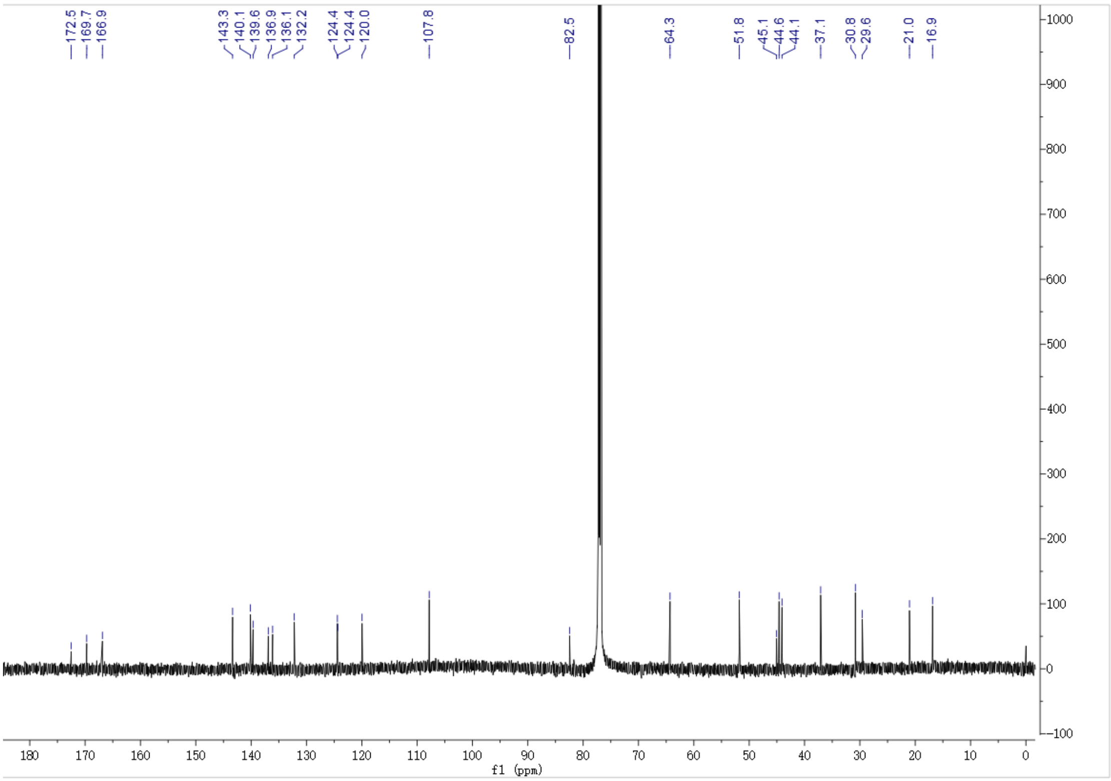

S23. ^{13}C NMR Spectrum of 4 in $CDCl_3$

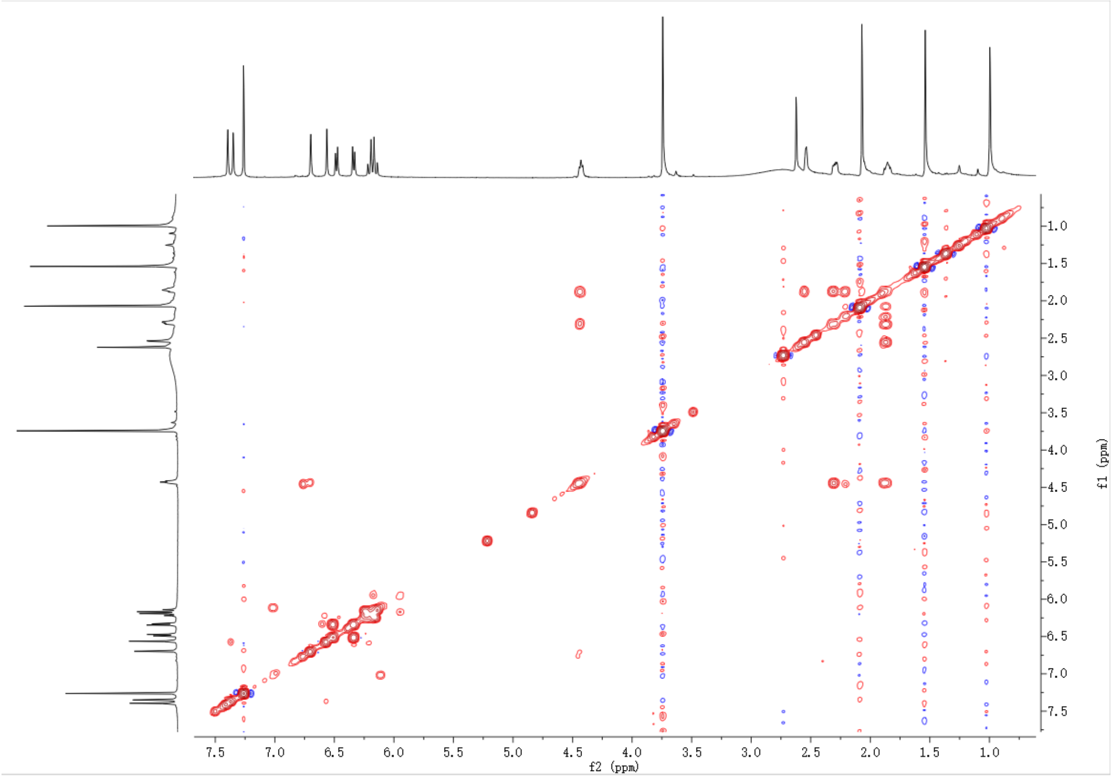

S24. ^{1}H-^{1}H COSY Spectrum of 4 in $CDCl_3$

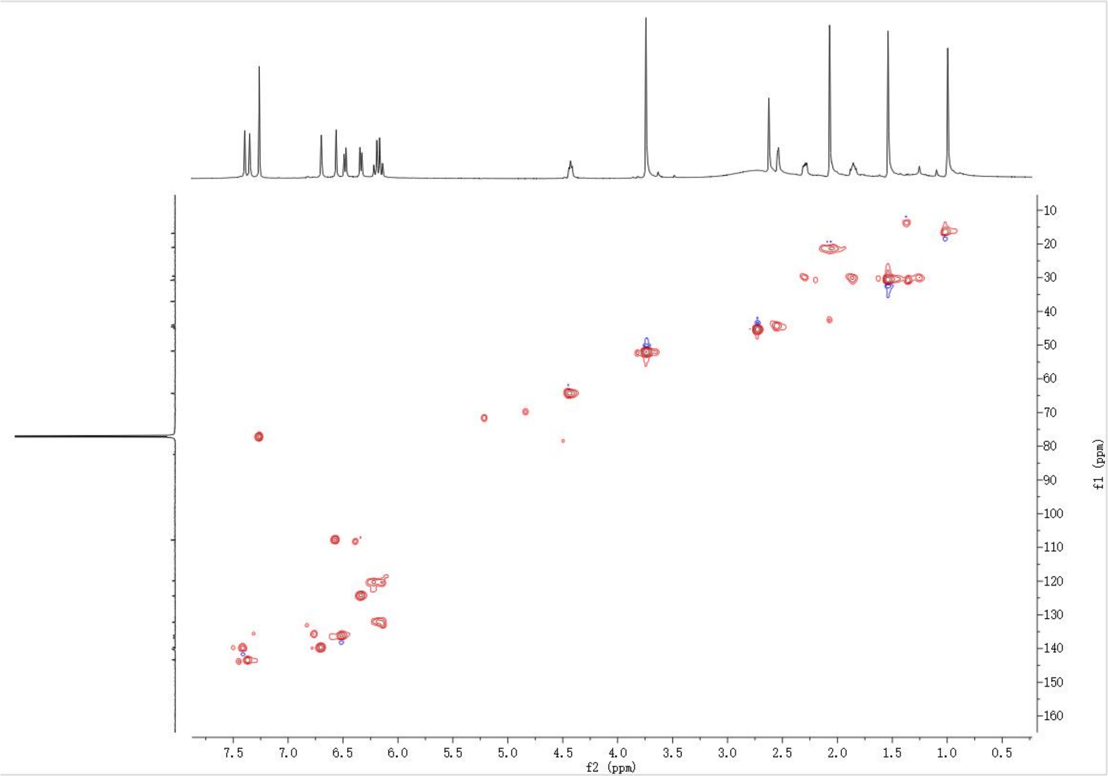

S25. HSQC Spectrum of 4 in $CDCl_3$

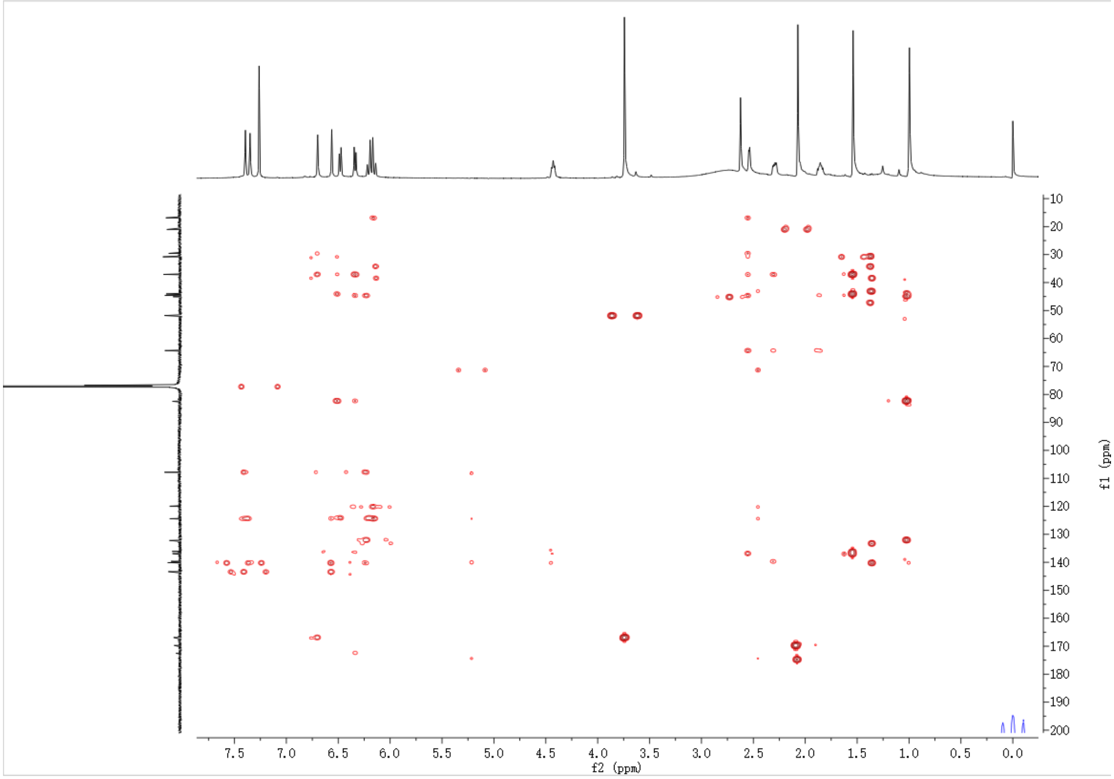

S26. HMBC Spectrum of 4 in $CDCl_3$

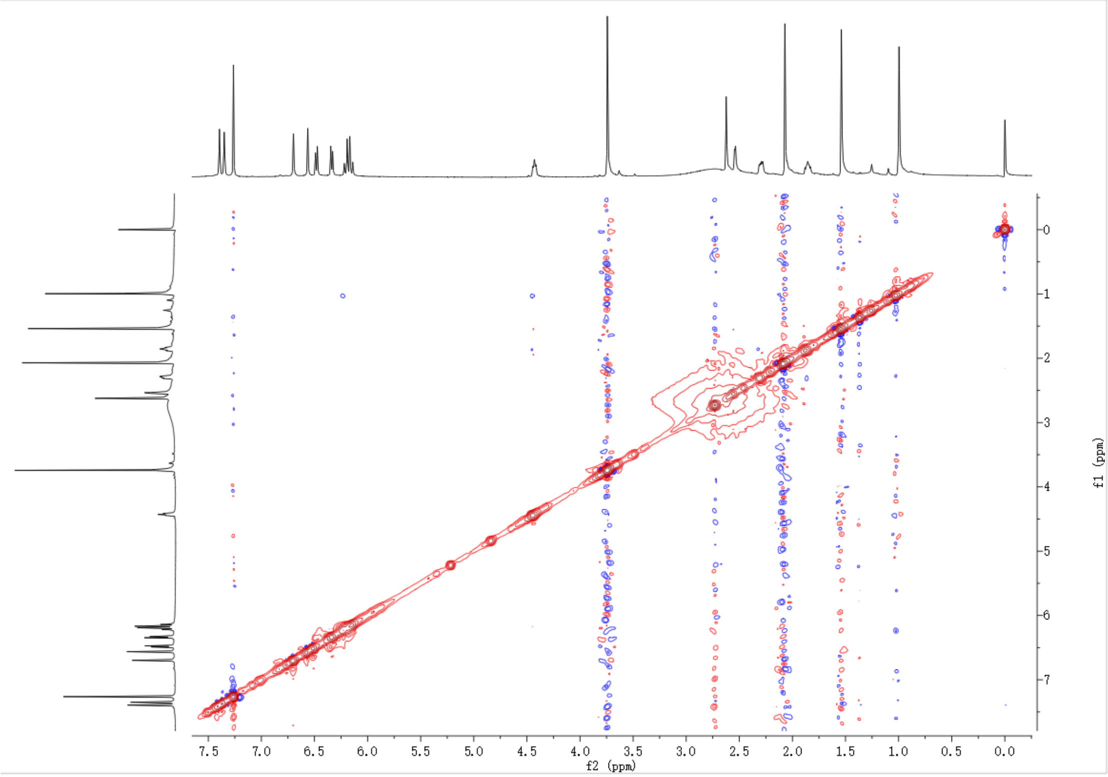

S27. NOESY Spectrum of 4 in $CDCl_3$

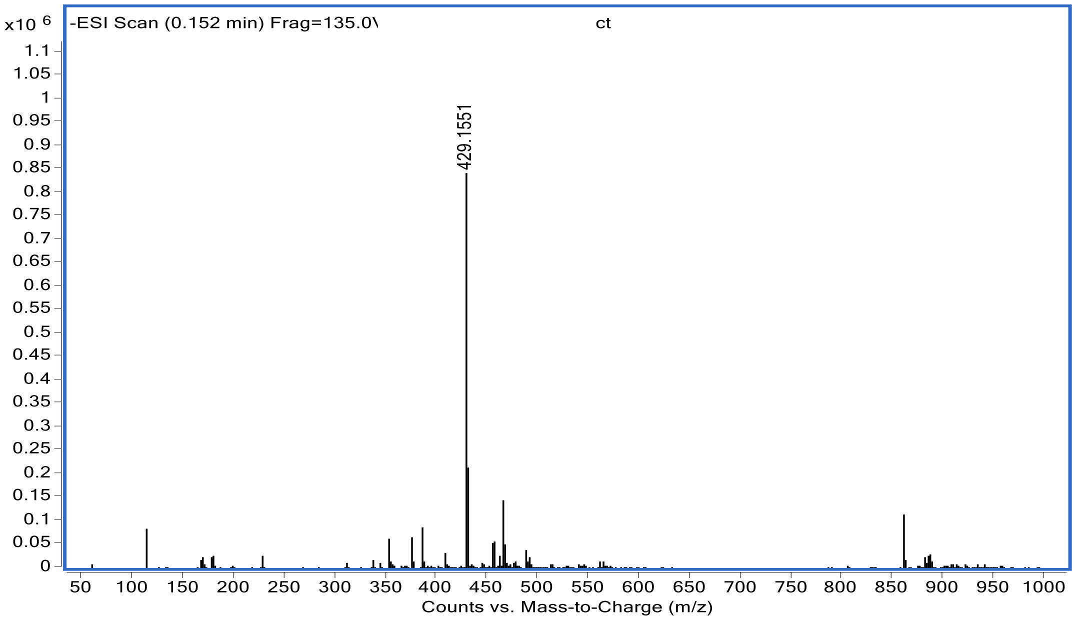

S28. HRESIMS Spectrum of 4

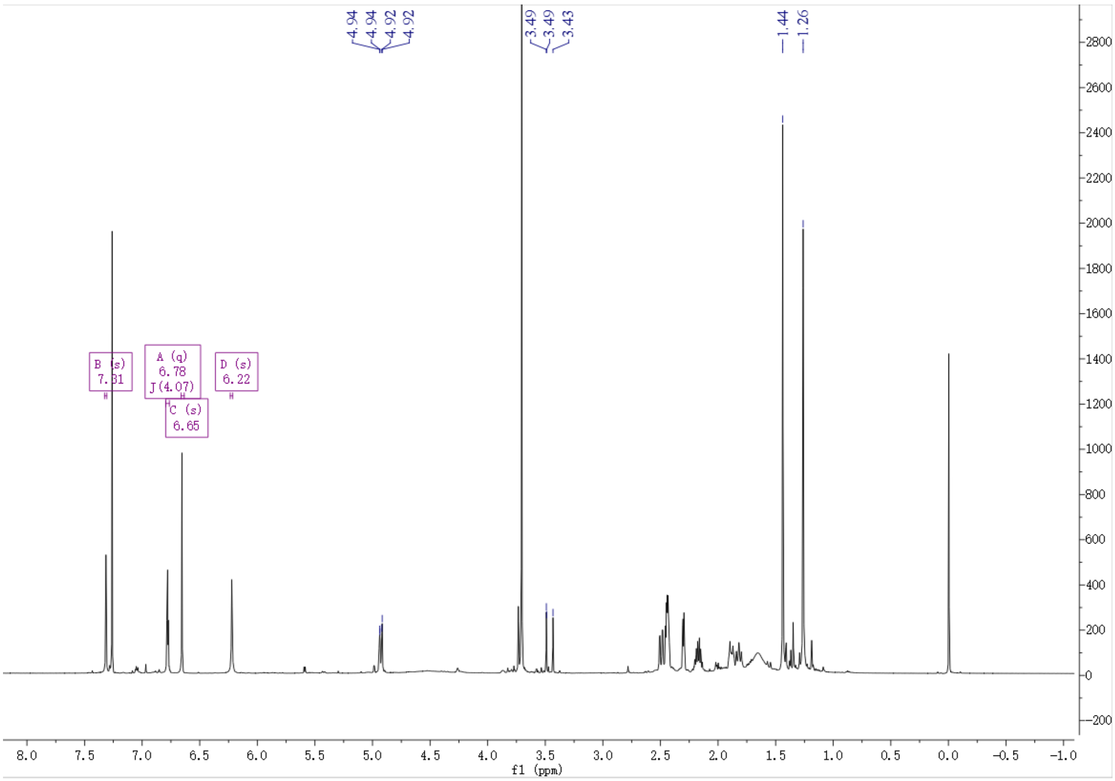

S29. ^{1}H NMR Spectrum of 5 in $CDCl_3$

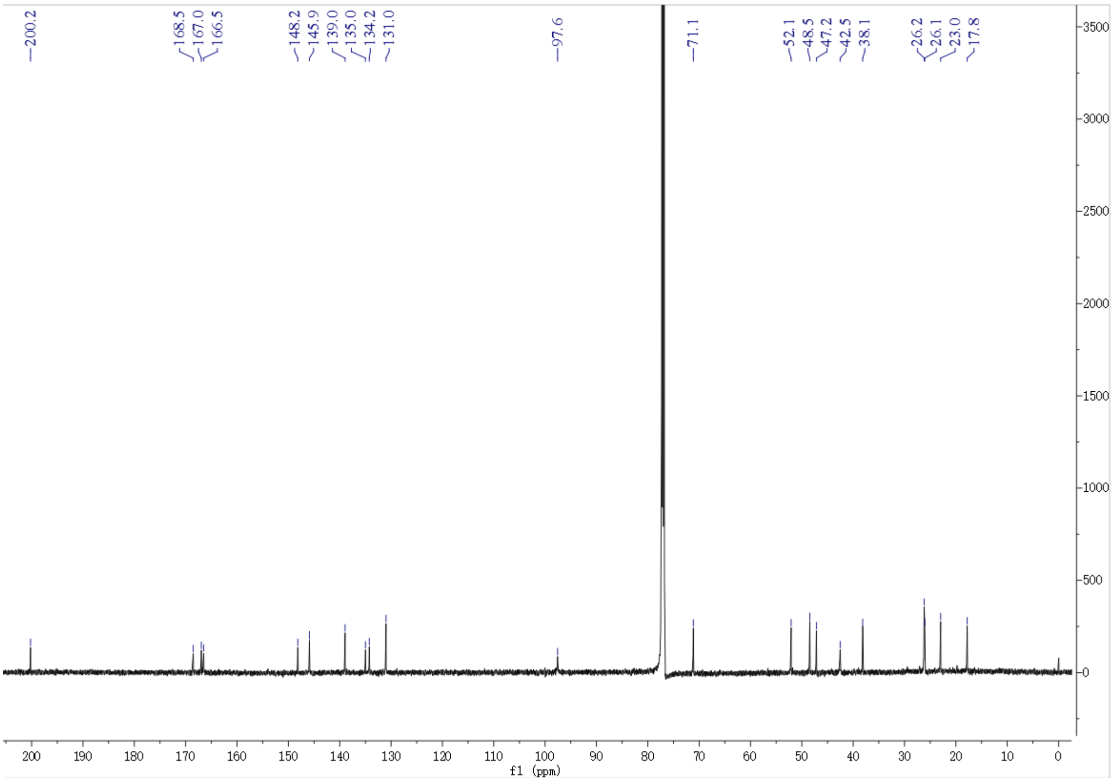

S30. ^{13}C NMR Spectrum of 5 in $CDCl_3$

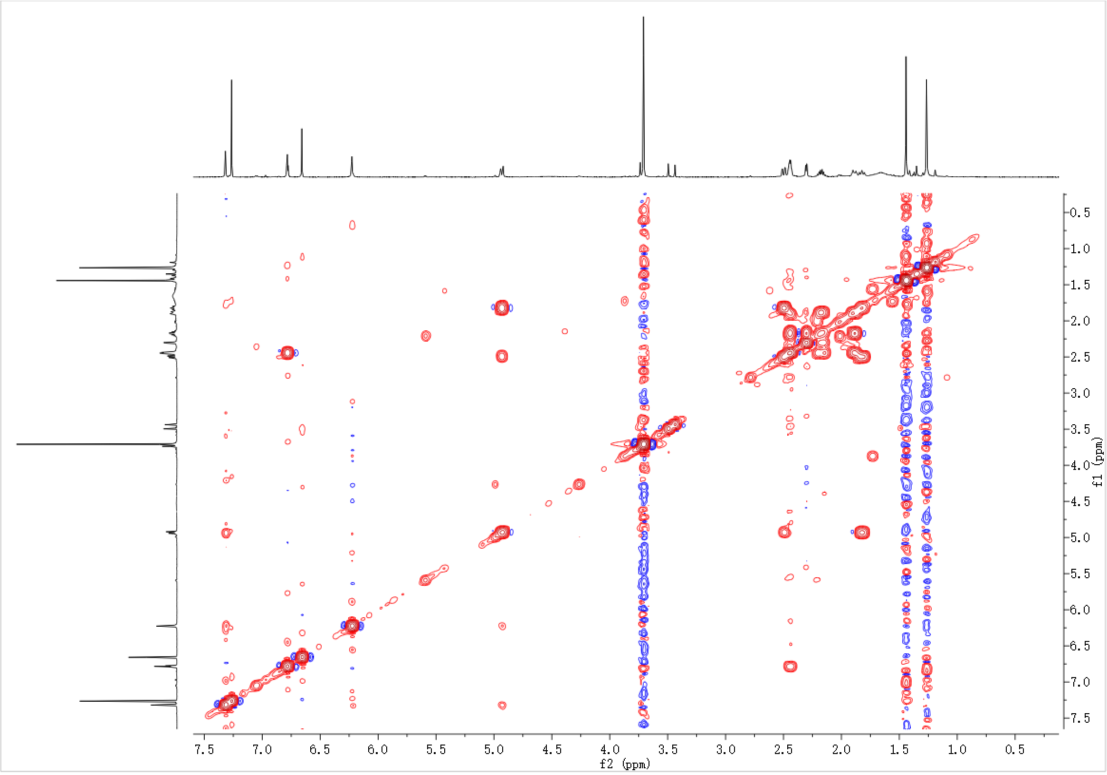

S31. ^{1}H-^{1}H COSY Spectrum of 5 in $CDCl_3$

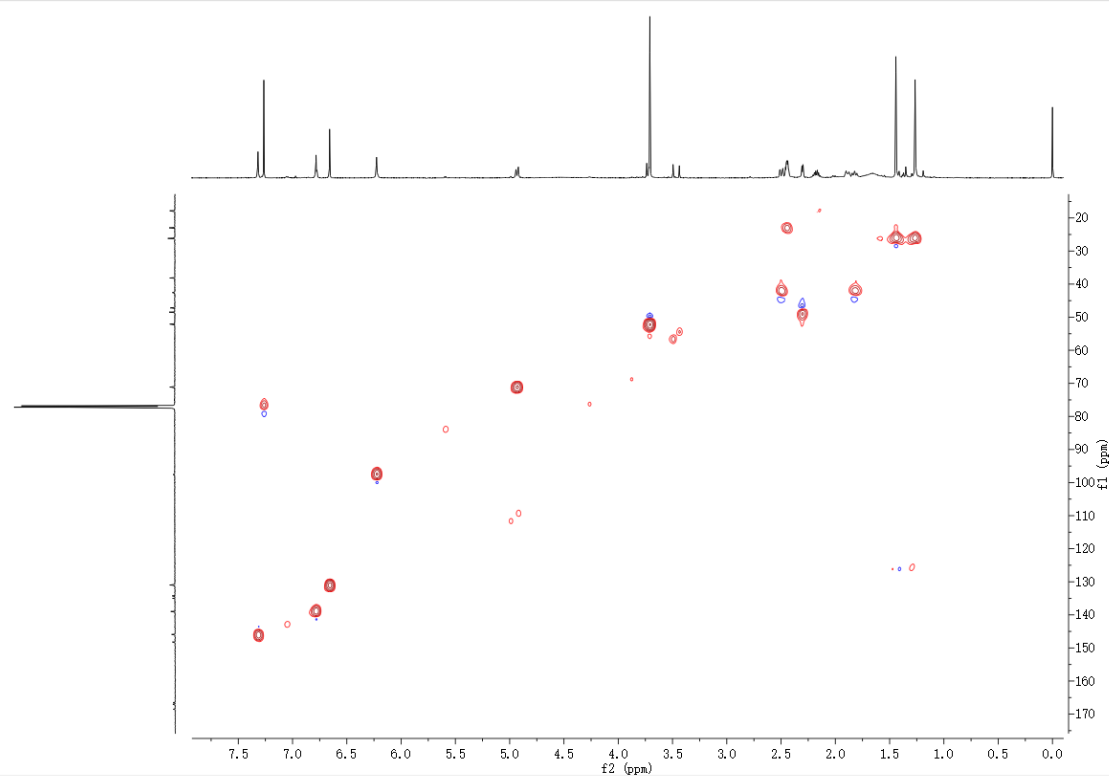

S32. HSQC Spectrum of 5 in $CDCl_3$

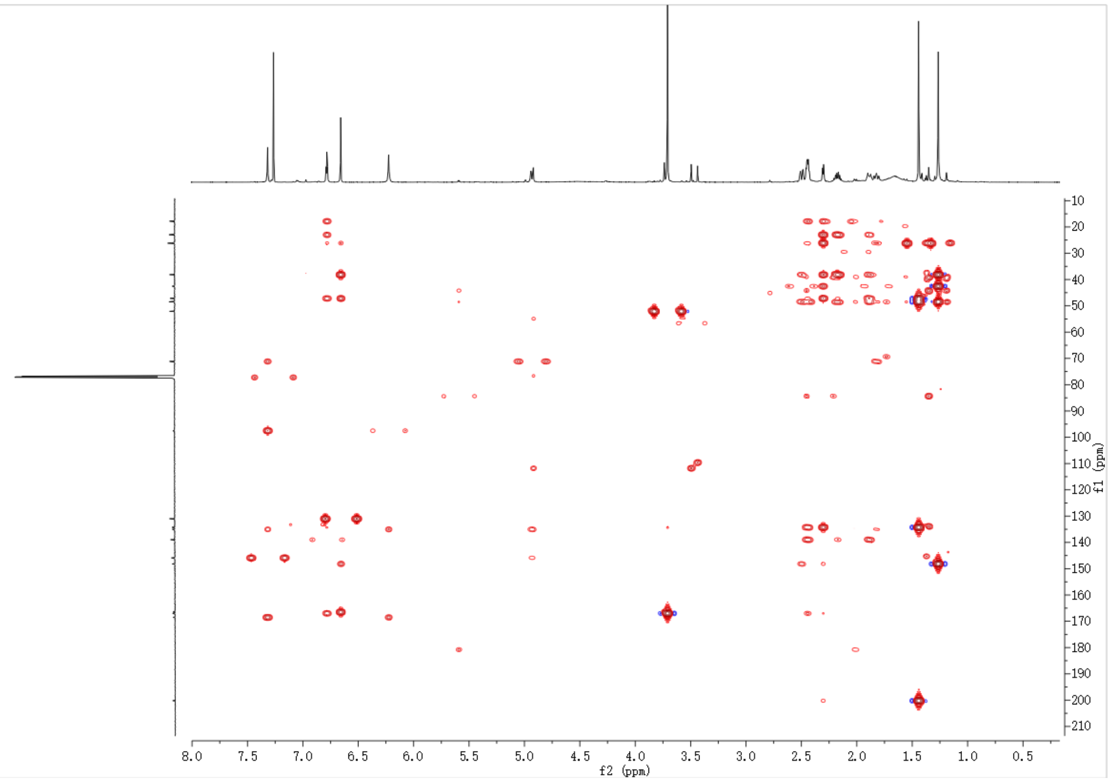

S33. HMBC Spectrum of 5 in $CDCl_3$

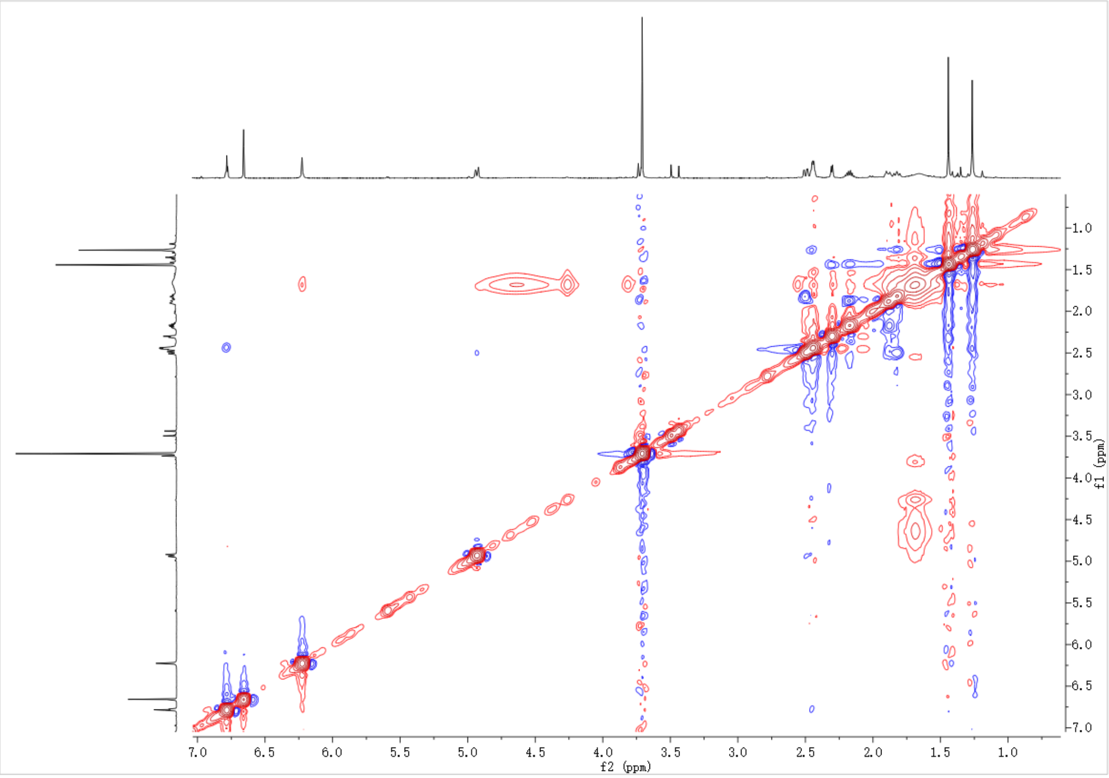

S34. NOESY Spectrum of 5 in $CDCl_3$

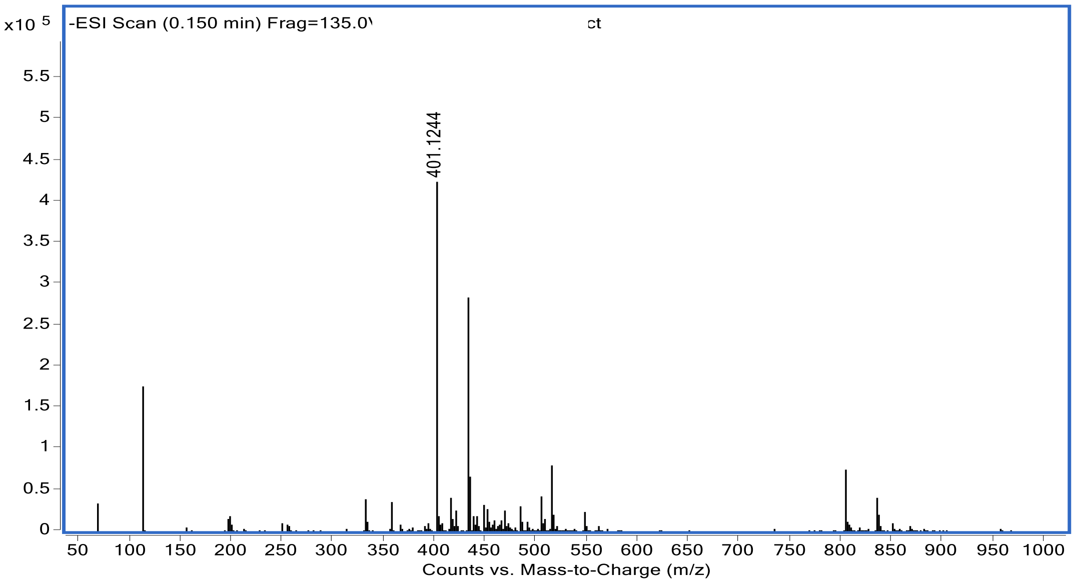

S35. HRESIMS Spectrum of 5

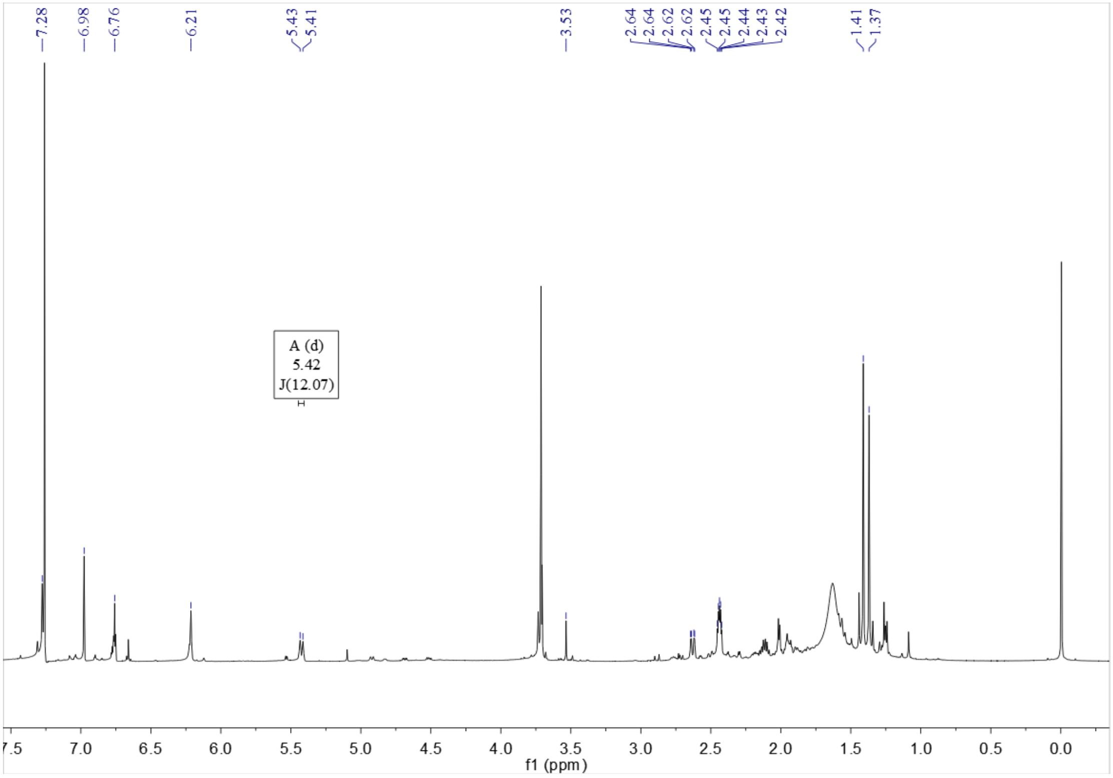

S36. ^{1}H NMR Spectrum of 6 in $CDCl_3$

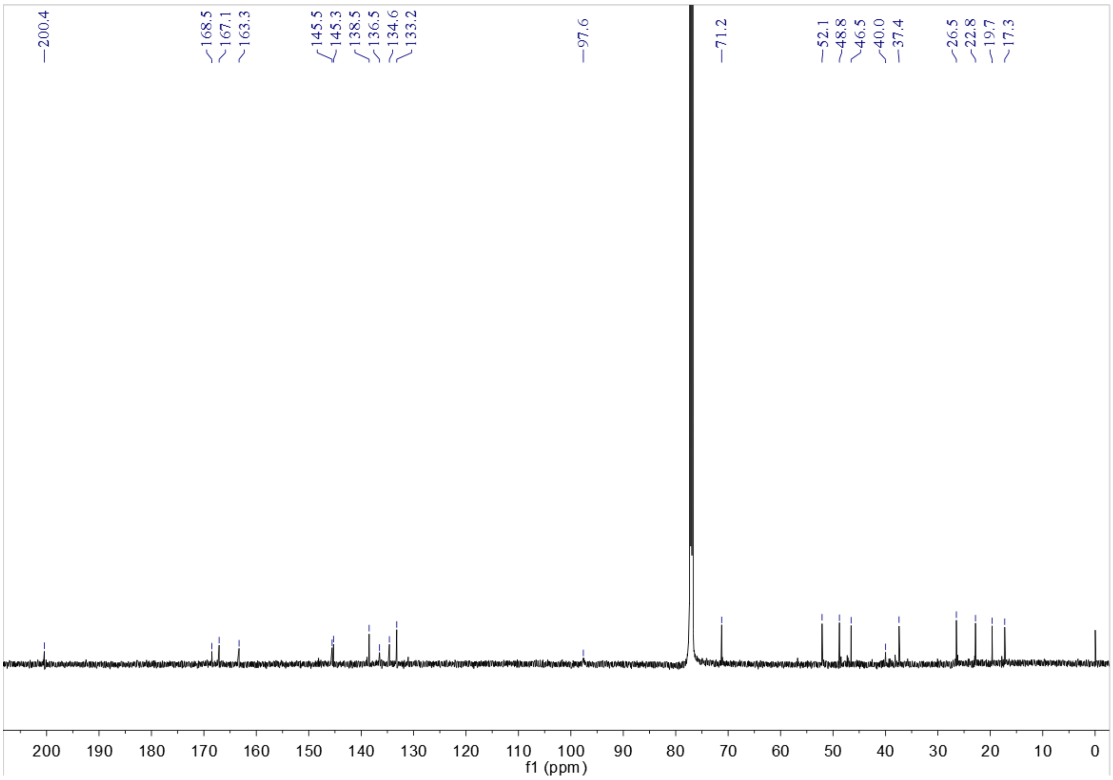

S37. ^{13}C NMR Spectrum of 6 in $CDCl_3$

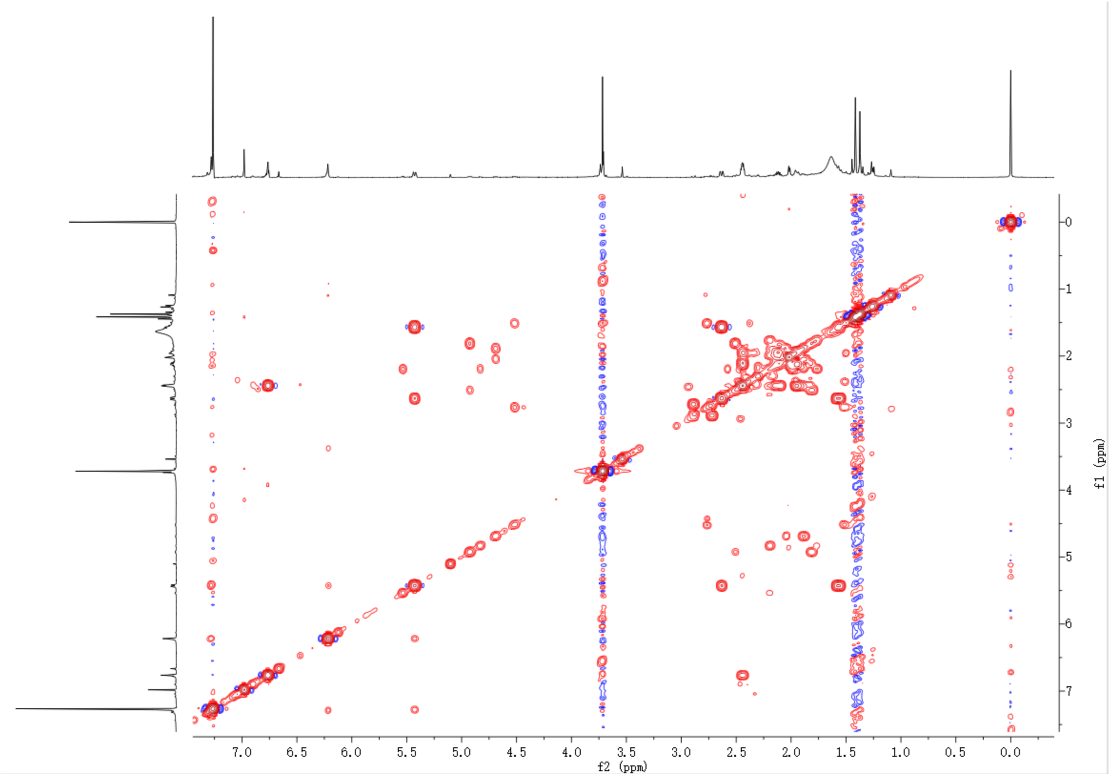

S38. ^{1}H-^{1}H COSY Spectrum of 6 in $CDCl_3$

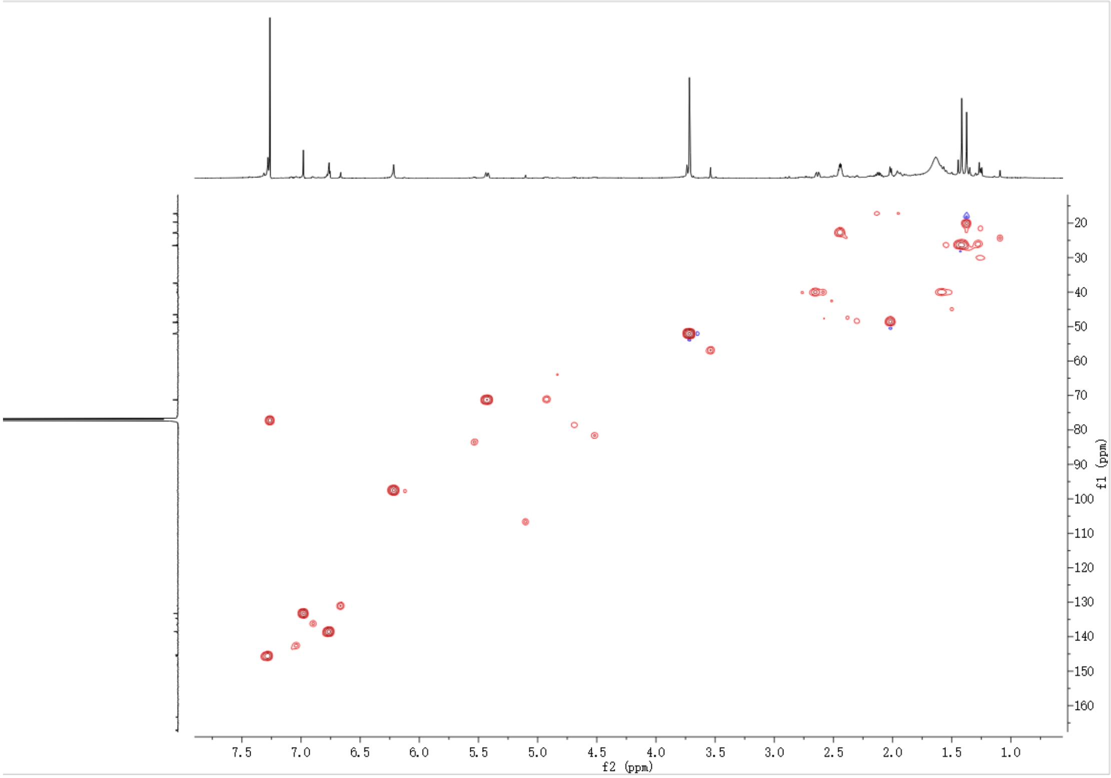

S39. HSQC Spectrum of 6 in $CDCl_3$

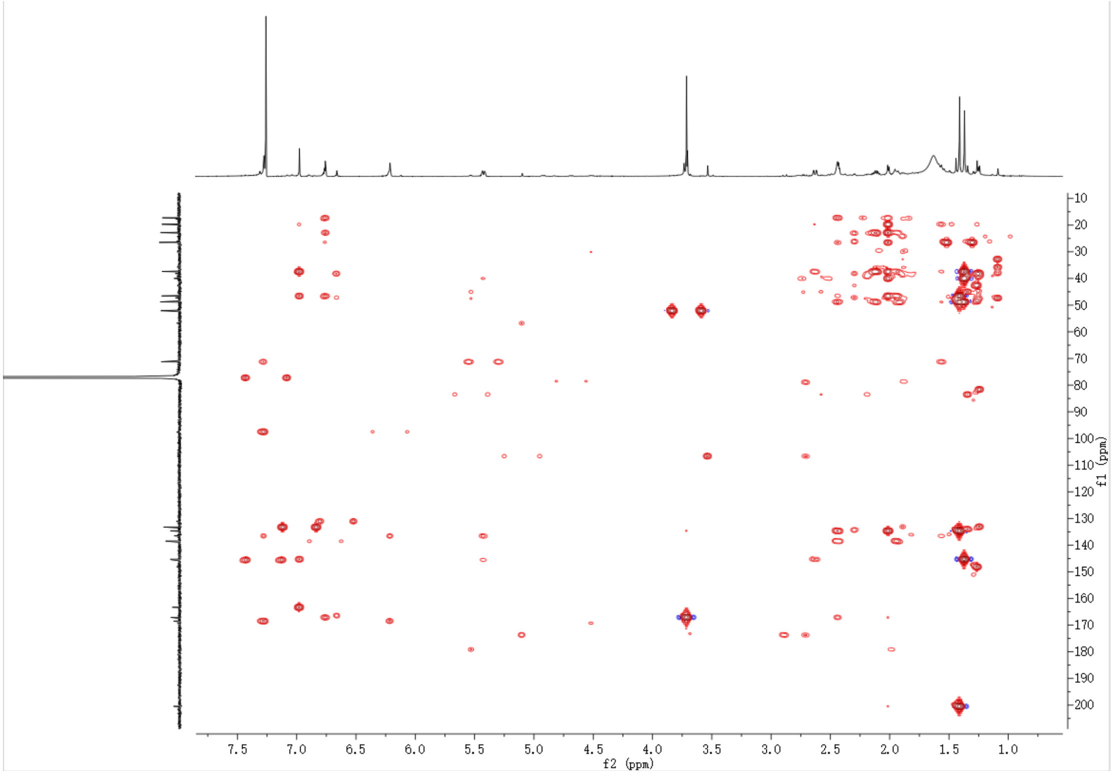

S40. HMBC Spectrum of 6 in $CDCl_3$

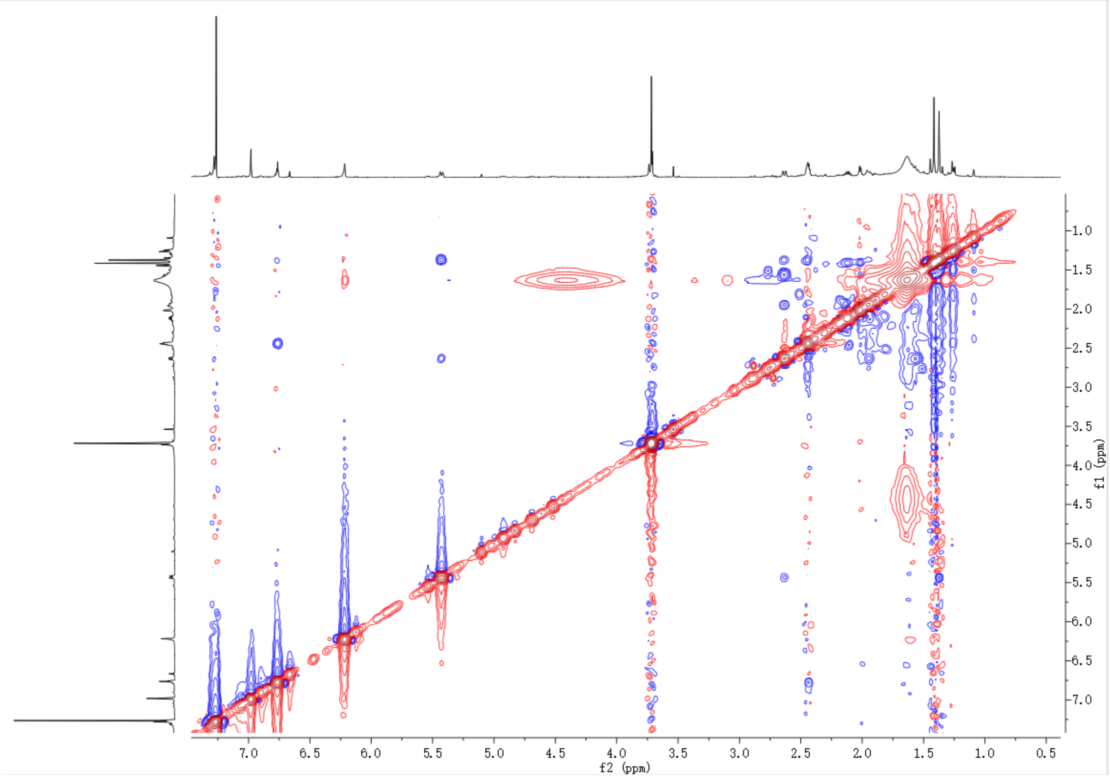

S41. NOESY Spectrum of 6 in $CDCl_3$

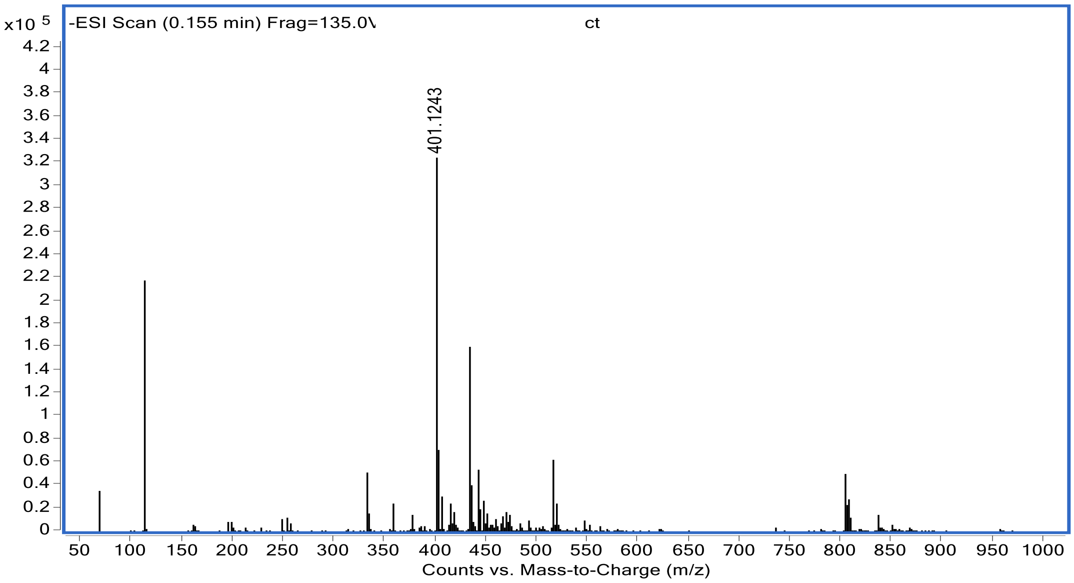

S42. HRESIMS Spectrum of 6

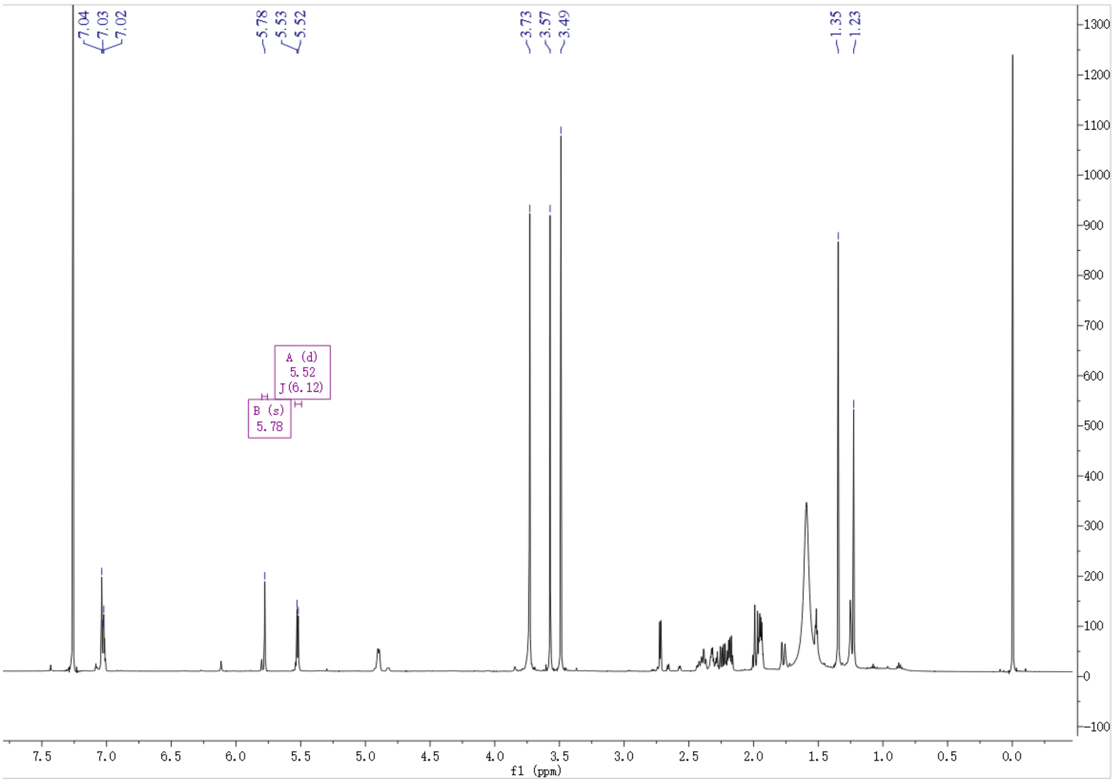

S43. ^{1}H NMR Spectrum of 7 in $CDCl_3$

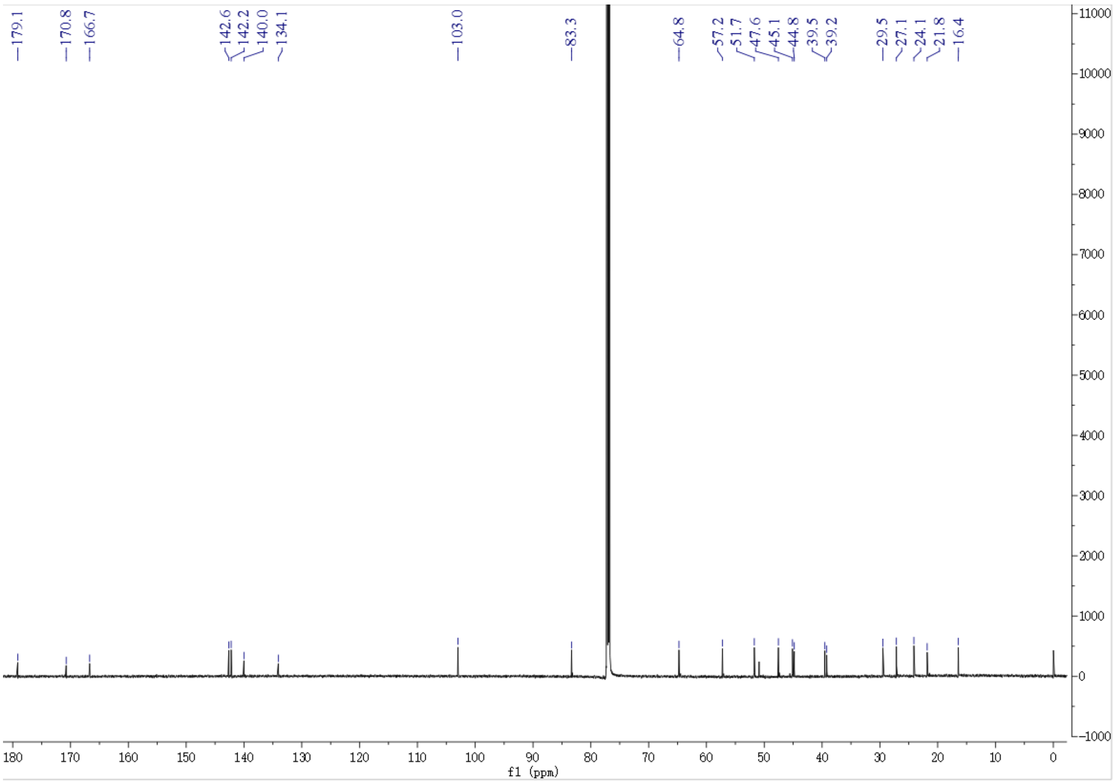

S44. ^{13}C NMR Spectrum of 7 in $CDCl_3$

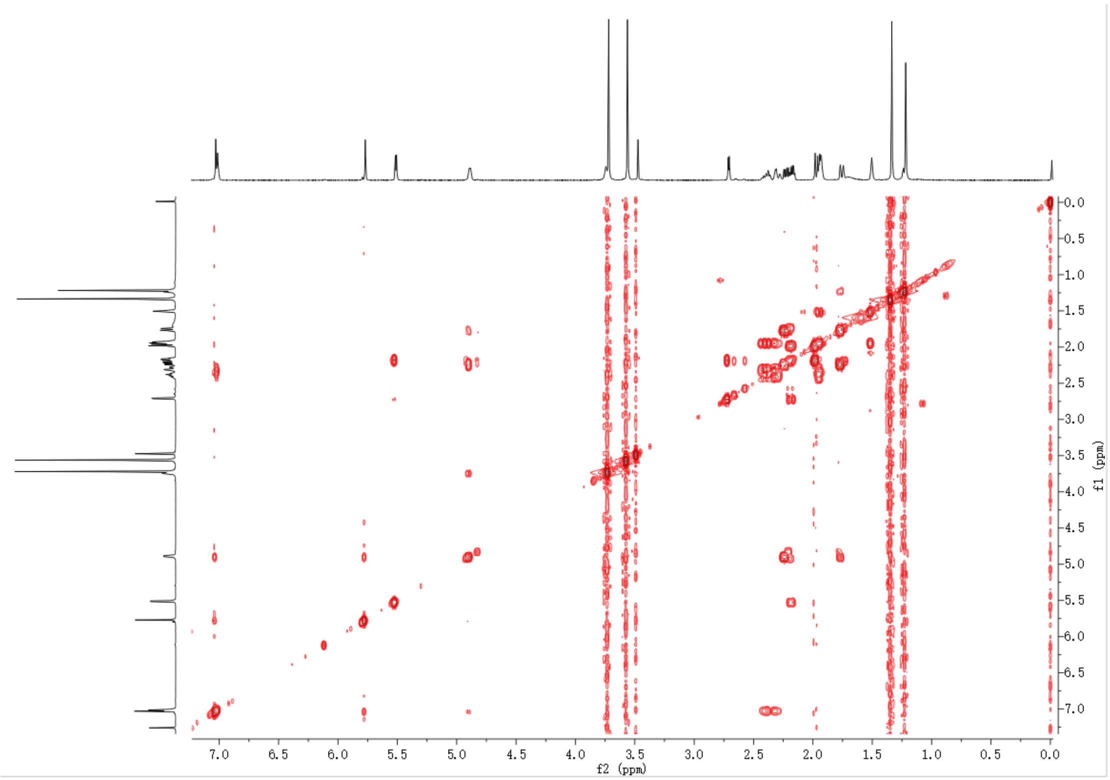

S45. ¹H-¹H COSY Spectrum of 7 in $CDCl_3$

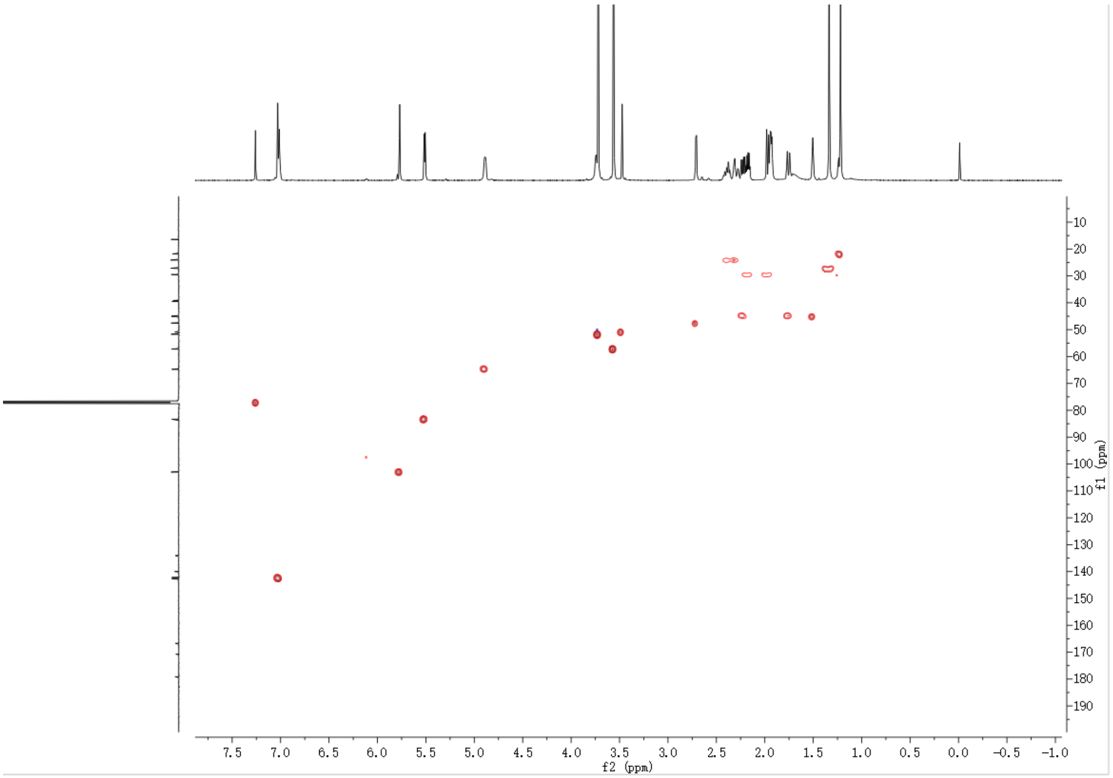

S46. HSQC Spectrum of 7 in $CDCl_3$

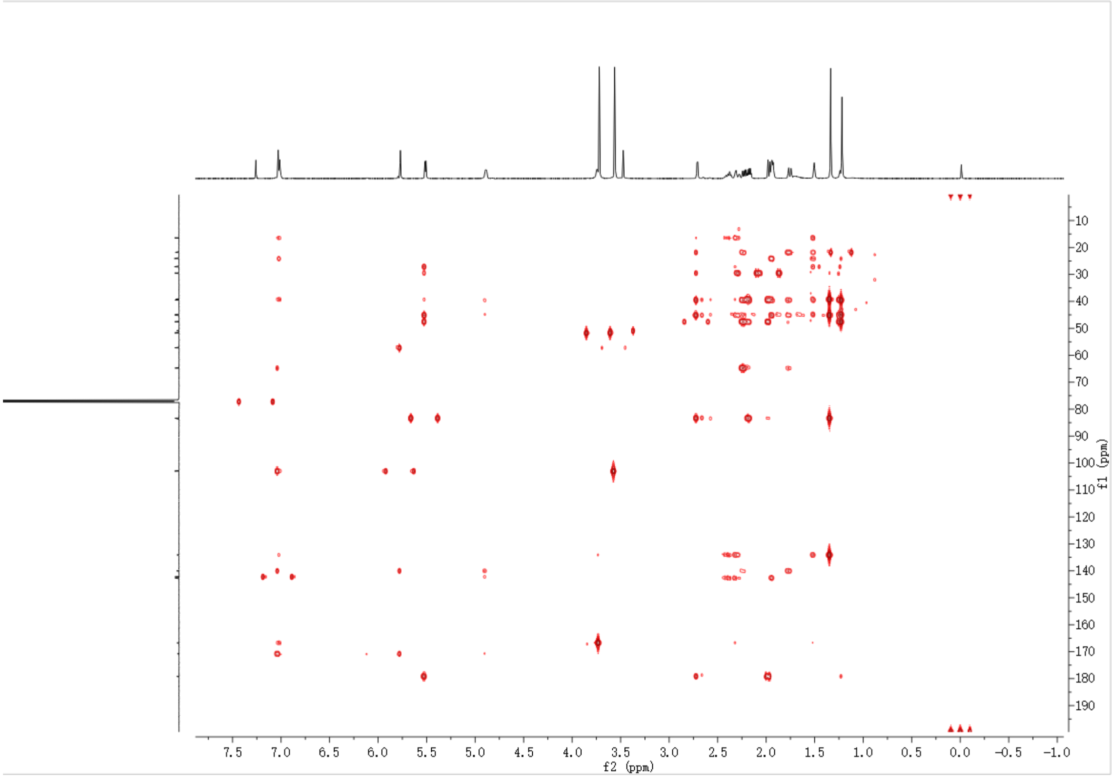

S47. HMBC Spectrum of 7 in $CDCl_3$

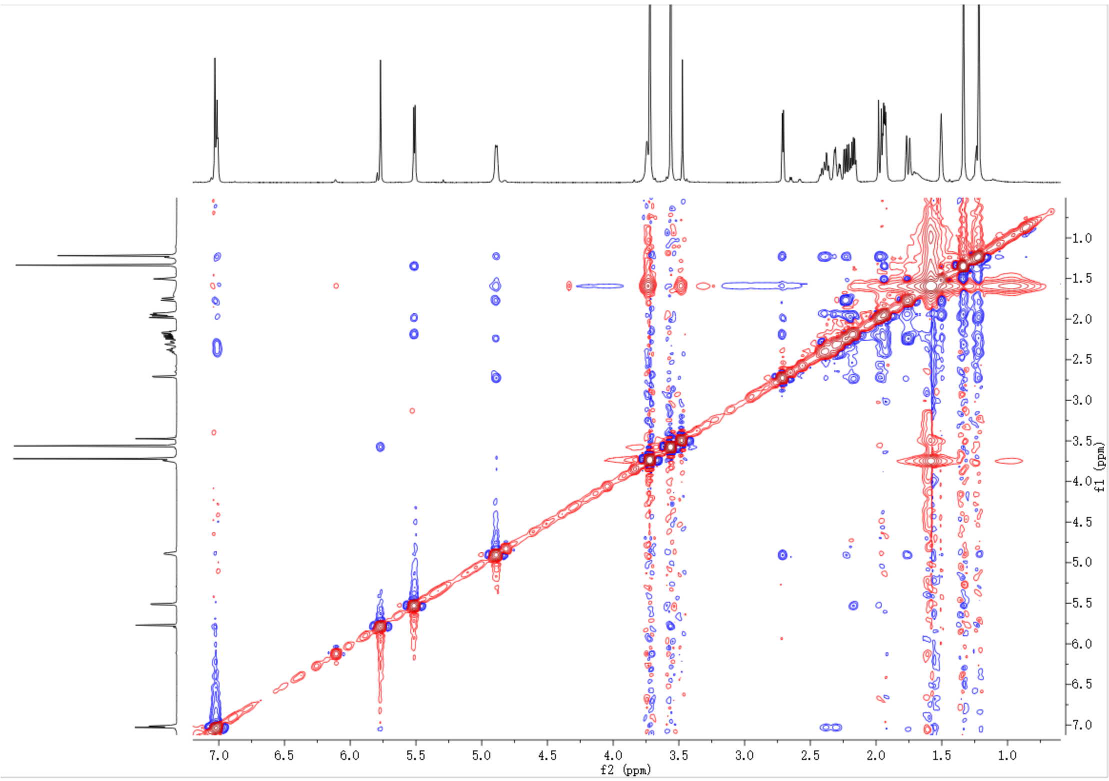

S48. NOESY Spectrum of 7 in $CDCl_3$

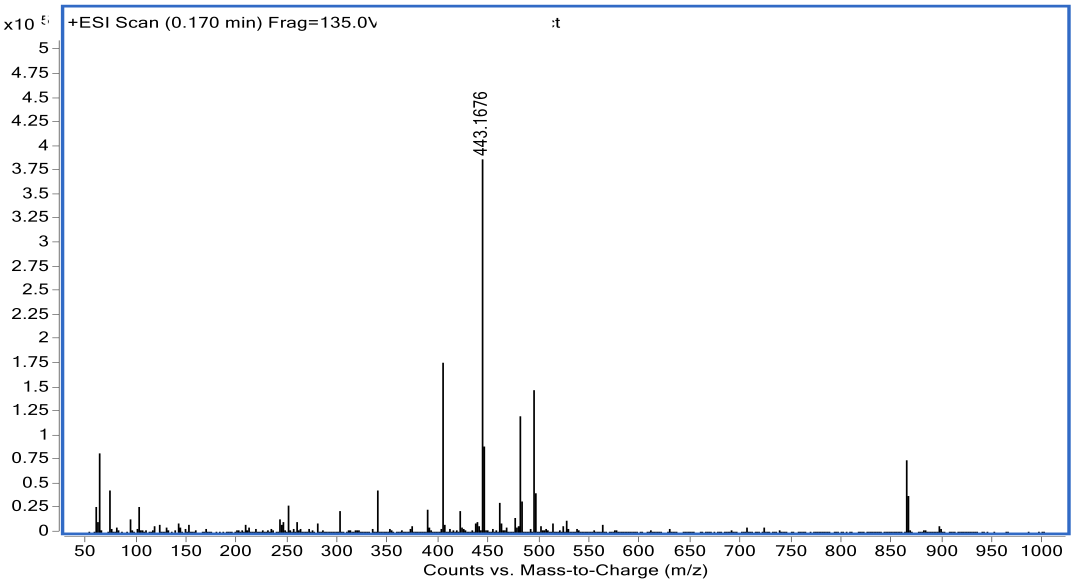

S49. HRESIMS Spectrum of 7
